# Genetic dissection and transcriptomic analysis of a novel high‐tillering phenotype in rice derived from weedy rice (Hapcheonaengmi3) and Tongil‐type Rice (Milyang23)

**DOI:** 10.1002/tpg2.70244

**Published:** 2026-04-21

**Authors:** Kyu‐Chan Shim, Donghyun Jeon, Yun‐A Jeon, Cheryl Adeva, Hyun‐Sook Lee, Ju‐Won Kang, Sa‐Eun Park, Sang‐Nag Ahn, Inkyu Park

**Affiliations:** ^1^ Department of Crop Science, College of Agriculture & Life Sciences Chungnam National University Daejeon South Korea; ^2^ Department of Biology and Chemistry Changwon National University Changwon South Korea

## Abstract

Rice (*Oryza sativa* L.) tillering is a critical determinant of grain yield, yet the genetic mechanisms underlying non‐productive tillers remain poorly understood. Here, we report a novel high‐tillering (HT) phenotype derived from a cross between the elite cultivar Milyang23 and weedy rice Hapcheonaengmi3. The HT phenotype was absent in parental lines, suggesting it arises from unique allelic interactions. To elucidate the genetic basis of HT, we conducted quantitative trait locus sequencing and linkage analysis using recombinant inbred lines and an F_2_ population. We identified and fine‐mapped two major loci, *qHT1* and *qHT6*, where alleles from Hapcheonaengmi3 induce the HT phenotype in the Milyang23 background. Advanced fine mapping facilitated by window‐size adjustments further resolved *qHT1* into two candidate intervals (*qHT1.1* and *qHT1.2*). Candidate gene analysis highlighted a putative *SQUAMOSA PROMOTER BINDING PROTEIN‐LIKE* (*SPL*) transcription factor within *qHT6*. Furthermore, transcriptomic profiling of HT plants uncovered extensive reprogramming of hormone signaling, specifically affecting the gibberellin, auxin, and cytokinin pathways. Notably, the *miR156–SPL* module, previously implicated in bushy tillering, exhibited significant expression changes, identifying it as a key regulatory candidate. These findings provide crucial insights into a novel polygenic mechanism controlling rice tillering and highlight the value of cryptic variation in weedy rice.

## Core Ideas

- A novel high‐tillering phenotype was discovered from an interaction between cultivated rice and weedy rice alleles.
- Quantitative trait locus sequencing and linkage analysis identified two major loci, *qHT1* and *qHT6*, controlling the high‐tillering trait.
- Transcriptome analysis revealed extensive hormonal signaling reprogramming involving the *miR156*–*SPL* regulatory module.
- The results demonstrate that cryptic genetic variation in weedy rice can generate new plant architecture traits useful for rice breeding.

## Plain Language Summary

Rice (*Oryza sativa* L.) yield depends heavily on the number of tillers, or branches, that a plant produces. In this study, we discovered a unique rice plant with an exceptionally high number of tillers. This “high‐tillering” trait appeared in the offspring of a cross between a cultivated rice and a wild‐like “weedy” rice, even though neither parent showed this characteristic. By analyzing their DNA, we pinpointed two specific genetic regions inherited from the weedy rice that interact to trigger this bushy growth. Our investigation revealed that these genetic changes significantly alter the plant's hormone balance, leading to excessive branching. This discovery is important because it demonstrates that weedy rice, often considered an agricultural weed, contains hidden genetic diversity. Unlocking these hidden traits can help scientists develop new rice varieties with improved structures and potentially higher yields to meet global food demands.

**Abbreviations**
CKX: cytokinin oxidase/dehydrogenase
DEGs: differentially expressed genes
DTE: dark‐tip embryo
*GNP6*
: grain number per panicle 6
GO: gene ontology
HT: high‐tillering
KEGG: Kyoto Encyclopedia of Genes and Genomes
log_2_FC: log_2_FoldChange
MOC1: MONOCULM 1
QTL: quantitative trait locus
RDA: Rural Development Administration
SNP: single‐nucleotide polymorphism
SPL: SQUAMOSA PROMOTER BINDING PROTEIN‐LIKE

## 1 INTRODUCTION

Rice (*Oryza sativa* L.) is one of the most important cereal crops, and most of the Asian countries consume it as a staple food (Kwak & Han, 2023; J.‐Y. Lee, Park, et al., 2024). Increasing rice production is necessary to meet the demands of the growing world population (G.‐M. Lee, Jang, et al., 2024). Plant architecture is one of the most important agronomic traits and is determined by factors such as tiller number and leaf angle (Yang & Hwa, 2008). Rice tiller is a key determinant of the number of panicles and grain yield and the number of productive tillers directly affects rice yield (Y. Lu et al., 2022). Many genetic studies have focused on increasing the number of productive tillers, while studies on non‐productive tillers remain. The genetic and molecular mechanisms underlying non‐productive tillers are still not well understood.

Rice tiller development is orchestrated by a complex interplay of genetic and hormonal factors (Y. Lu et al., 2022). Several key regulators of tillering have been identified, including *MONOCULM1* (*MOC1*), a GRAS family transcription factor required for axillary meristem initiation (Li et al., 2003). Alterations in *MOC1* function result in abnormal tiller development (Shao et al., 2019; Zhang et al., 2021). Similarly, *LAX PANICLE1/LAX2* and *APO* genes regulate meristem maintenance and influence tiller number and panicle architecture (Ikeda‐Kawakatsu et al., 2009; Ikeda‐Kawakatsu et al., 2012; Oikawa & Kyozuka, 2009; Tabuch et al., 2011).

Tillering is also regulated by hormonal pathways, particularly auxin, cytokinin, and gibberellin (GA) (Miura et al., 2010). Auxin suppresses axillary bud outgrowth through regulators such as *OsTB1* (Takeda et al., 2003), whereas cytokinins promote bud activation, with *OsCKX2* modulating cytokinin levels and influencing tiller number and grain yield (Ashikari et al., 2005). Additional regulators such as *plant architecture and yield 1* affect auxin transport and contribute to plant architecture (Zhao et al., 2015). The *ideal plant architecture 1* gene encoding a *SQUAMOSA PROMOTER BINDING PROTEIN‐LIKE* (*SPL*) transcription factor suppresses *TB1*, simultaneously promoting panicle branching (Jiao et al., 2010; Z. Lu et al., 2013; Miura et al., 2010). The balance between these genetic and hormonal factors is crucial for optimizing rice plant architecture to maximize yield.

Non‐productive tillers, which do not contribute to grain production, are an important consideration in tillering regulation. The *Bushy dwarf tiller 1* mutant was identified from a DNA transposon *nDart1* insertion line in which the transposon was inserted upstream of the *miR156d* precursor gene (Os02g0180800) (Hayashi‐Tsugane et al., 2015). The *nDart1* insertion led to an increased transcription level of *miR156d*, resulting in semi‐dwarf and bushy tillering phenotypes. The bushy tillering phenotype was generated by the development of secondary branches from irregular positions on culms (Hayashi‐Tsugane et al., 2015). Another microRNA *miR529a* also regulates plant architecture including plant height, tiller number, panicle architecture, and grain size through *SPL* gene regulation in rice (Yan et al., 2021). The *miR529a* was a highly expressed gene in seedling and panicle, and showed highly similar sequences with *miR156* (Yue et al., 2017). In addition, *miR156* and *miR529a* shared common target *SPL* genes *OsSPL2*, *OsSPL14*, *OsSPL16*, *OsSPL17*, and *OsSPL18*. These reports indicate that the SPL gene family and microRNAs are strongly associated with tiller development.

Phenotypic traits are the expression of genes in an observable way, and these characteristics are controlled by genes. Both genes and phenotypic traits are inherited from parents, and most plant genetic studies have been conducted to identify genes or loci that control phenotypic traits observed in one of the parental lines (Tanksley, 1993). However, some traits are observed only in the progeny and not in their parental lines, such as hybrid weakness syndromes. For example, a hybrid weakness phenotype was observed at two loci, *Hwi1* and *Hwi2*, in a pyramiding *japonica* genetic line, with *Hwi1* and *Hwi2* introgressed from wild rice (*Oryza rufipogon*) and an indica allele, respectively (Chen et al., 2014). The pyramiding of these two loci resulted in short stature and impaired root formation, whereas the parental lines did not exhibit these weakness phenotypes (S. H. Kim et al., 2022) reported a dark‐tip embryo (DTE) phenotype as a hybrid weakness syndrome. The *DTE9* locus, derived from the *O. rufipogon* (W1944) allele, caused the DTE phenotype in the Korean *japonica* rice cultivar Hwayeong genetic background. Interestingly, the DTE phenotype was not observed in either of the parental lines (Hwayeong and W1944). These hybrid weakness phenotypes occurred due to the interaction of more than two loci contributed by each parent.

In this study, we identified a novel high‐tillering (HT) phenotype, which produced tillers at a higher internode than normal tillers and exhibited leafy, continuous nodes in the introgression line CR40. This HT phenotype has not been previously reported in cultivated or weedy rice, suggesting it is a newly arisen trait resulting from the unique allelic combination in the introgression line. CR40 was derived from a cross between the Tongil‐type (*Japonica/Indica*) Korean elite line Milyang23 and weedy rice Hapcheonaengmi3. Interestingly, the HT phenotype was not detected in either Milyang23 or Hapcheonaengmi3, indicating that the allelic combination of the two parents generated HT. Quantitative trait locus sequencing (QTL‐seq) was employed to identify genomic regions associated with the HT phenotype, and two putative QTLs were detected on chromosomes 1 and 6. To validate and refine these QTLs, we developed an F_2_ population by crossing HT35, which exhibited a stronger HT phenotype than CR40, with Milyang23. Two QTLs were confirmed on chromosomes 1 and 6, and the loci were narrowed down to 418 and 106 kb, respectively. These results indicate that two loci on chromosomes 1 and 6 from the Hapcheonaengmi3 allele contributed to the HT phenotype in the Milyang23 genetic background. To gain further insight into the molecular mechanism underlying the HT phenotype, transcriptome analysis was conducted.

**Core Ideas**

- A novel high‐tillering phenotype was discovered from an interaction between cultivated rice and weedy rice alleles.
- Quantitative trait locus sequencing and linkage analysis identified two major loci, *qHT1* and *qHT6*, controlling the high‐tillering trait.
- Transcriptome analysis revealed extensive hormonal signaling reprogramming involving the *miR156*–*SPL* regulatory module.
- The results demonstrate that cryptic genetic variation in weedy rice can generate new plant architecture traits useful for rice breeding.

## 2 MATERIALS AND METHODS

### 2.1 Plant materials and phenotypic characterization

#### 2.1.1 Plant materials

In our previous study, a total of 68 introgression lines were developed from a cross between Milyang23 (*japonica/indica*) and Hapcheonaengmi3, and two parental lines were provided from the Rural Development Administration (RDA, South Korea) (Figures S1 and S2) (Oh et al., 2010). Among these 68 lines, only CR40 exhibited an HT phenotype and was selected to elucidate its genetic basis. For genetic mapping, CR40 was backcrossed with Milyang23, and 49 F_9_ lines (HT3–51) were generated using the single seed descent method. The HT3–51 lines were used for primary QTL mapping using a QTL‐seq approach. Among the 49 lines, we selected HT35, which displayed a strong and stable HT phenotype, and HT35 was crossed with Milyang23 to develop a population for QTL confirmation and validation (Figure S3). A total of 203 F_2_ plants were generated for QTL validation and fine‐mapping (Figure S1). Plants were grown under field conditions at Chungnam National University experimental fields (Daejeon, Republic of Korea). Transplanting was performed in late May with a spacing of 15 cm between plants within rows and 30 cm between rows, following standard agronomic practices. Fertilizer application, irrigation, and pest management were conducted according to RDA cultivation guidelines (RDA, 2012). Phenotypic evaluation was conducted at the post‐flowering stage when high‐internode tiller emergence was clearly distinguishable.

#### 2.1.2 HT phenotype evaluation for mapping two populations

The HT phenotype was classified based on distinct morphological characteristics rather than overall plant appearance. Specifically, HT plants were defined by (i) emergence of tillers from upper internodes after flowering, (ii) repeated node formation above the conventional basal tillering zone, and (iii) activation of secondary tillers at elevated stem positions. Scoring was performed at the post‐flowering stage when these structural features became clearly visible.

For the primary QTL mapping, phenotyping was conducted from the F_4_ to F_9_ generations using 49 lines of the HT population. The phenotype was evaluated using a categorical scale of 1 (no HT, Milyang23 type), 2 (ambiguous), and 3 (clear HT, CR40 type). For the F_2_ population, HT severity was scored on a scale from 1 (no HT, Milyang23 type) to 9 (severe HT, HT35 type) to capture gradation in tillering intensity. Although categorical scoring was applied, classification was consistently based on the defined morphological criteria described above.

### 2.2 Genetic mapping and genomic analysis

#### 2.2.1 DNA extraction and genotype analysis

Fresh leaves were harvested for genomic DNA extraction, and the CTAB method was employed with minor modifications (W.‐J. Kim et al., 2020). PCR was performed following the method described by Shim et al. (2019). PCR products were separated on a 3% agarose gel stained with StaySafe Nucleic Acid Gel Stain (RBC). Genotyping was performed to confirm and validate the two QTLs identified through the QTL‐seq analysis. InDel markers for these chromosomal regions were designed based on polymorphisms between Milyang23 and Hapcheonaengmi3, as revealed by the QTL‐seq results. The primers used for QTL confirmation and validation are listed in Table S1.

#### 2.2.2 QTL analysis

For QTL‐seq analysis, two DNA bulks (HT group and no HT group) were generated based on the HT phenotype score. Eight lines were categorized into the HT group, and 12 lines were classified into the non‐HT group. Bulked DNAs were used to construct sequencing libraries using the TruSeq DNA PCR‐free Sample Preparation Kit (Illumina Inc.). Paired‐end sequencing was performed using the HiSeq‐X platform (Illumina Inc.). QTL‐seq analysis was conducted using the QTL‐seq pipeline (ver. 2.1.3) with default parameters (Takagi et al., 2013). Raw sequencing reads were trimmed using the Trimmomatic software included in the QTL‐seq pipeline. The trimmed reads were mapped to the rice reference genome (IRGSP‐1.0), and variants were identified. Single‐nucleotide polymorphism (SNP) and delta‐SNP indices were calculated, and a sliding window approach was employed to compute the average SNP and delta‐SNP indices with a 2 Mb window size and 100 kb increments. Based on the average SNP and delta‐SNP indices, a circos plot was generated using shinyCircos‐V2.0 with SNP indices of two bulked sequencing data and delta‐SNP index from sliding window analysis (Wang et al., 2023). A QTL was identified when the average delta‐SNP index was significantly greater than the surrounding region and exhibited an average *p* < 0.05.

#### 2.2.3 Whole‐genome resequencing and variant calling

Whole‐genome resequencing was performed on two rice cultivars, Milyang23 and Hapcheonaengmi3, to analyze genetic variations within promoter and genic regions of the candidate genes. Sequencing libraries were constructed using the TruSeq Nano DNA Kit (Illumina), and paired‐end sequencing was conducted on the Illumina NovaSeq X Plus platform. Raw sequencing data were subjected to quality control using FastQC (v0.11.8) and Trimmomatic (v0.38) to remove low‐quality reads and adapters (Bolger et al., 2014). The high‐quality reads were then aligned to the *Oryza sativa* reference genome (Nipponbare‐Reference‐IRGSP‐1.0) using BWA‐MEM (v0.7.17) (Li, 2013). For variant analysis, duplicate reads were marked and removed using Sambamba (v0.6.8) (Tarasov et al., 2015), and variant calling was performed using SAMtools and BCFtools (Danecek et al., 2021). The identified variants (SNPs and indels) were annotated using SnpEff (v4.3t) to predict their functional effects. Visual inspection of genetic variations was carried out using the integrative genomics viewer and illustrator for biological sequences (Liu et al., 2015; Robinson et al., 2011). Additionally, protein structures affected by non‐synonymous mutations were predicted using AlphaFold2 (Jumper et al., 2021).

To refine the candidate genomic regions associated with the HT phenotype, sliding window analysis was conducted within the QTL‐detected regions using a custom Python script (Supporting Information Data S1). Only SNPs with statistically significant SNP index differences (*p* < 0.01) were selected for the analysis to increase resolution and reduce background noise. Multiple window sizes and step increments were tested, starting from 2 Mb windows with 100 kb steps and progressively narrowing the intervals. Based on performance evaluation, the final analysis was performed using a 10 kb window (0.01 Mb) with a 5 kb step size. For each window, the mean value of the delta‐SNP index and the number of SNPs were calculated. Windows containing at least one significant SNP were retained for downstream visualization and interpretation. This high‐resolution approach enabled the precise dissection of QTL intervals based on their physical positions and facilitated the identification of sub‐regions potentially associated with the HT trait.

For linkage analysis, polymorphic InDel markers located within the QTL‐seq‐identified regions on chromosomes 1 and 6 were selected. A total of 41 markers were used to genotype the F_2_ population consisting of 204 individuals, including 17 markers on chromosome 1 and 24 markers on chromosome 6. Linkage maps were constructed using IciMapping 4.1 based on recombination frequencies among markers with a step size of 1 cM (Meng et al., 2015). The resulting linkage groups spanned 26.6 cM for chromosome 1 and 24.0 cM for chromosome 6. For QTL confirmation and validation using F_2_ population, IciMapping 4.1 software was used with additional inclusive composite interval mapping using the default parameters as follows: Step = 1 cM, PIN (phenotype on marker variables) = 0.001, and logarithm of odds (LOD) = 5 (Meng et al., 2015). The HT score was used as the phenotypic trait for QTL analysis.

### 2.3 Transcriptome analysis

#### 2.3.1 RNA extraction and transcriptome analysis

Total RNA was isolated from high tillers of HT35, flag leaves of Milyang23, internode of HT35, and internode of Milyang23 with three biological replications using RNAiso Plus following the manufacturer's instructions (TaKaRa). RNA‐seq libraries were prepared using the Illumina TruSeq RNA Sample Preparation Kit, following the manufacturer's guidelines (Illumina Inc.). Sequencing was performed on an Illumina HiSeq X platform, and raw reads were processed using Trimmomatic and BBDuk to eliminate adapters, low‐quality reads, and contaminants (Bolger et al., 2014; Bushnell, 2014). The filtered reads were aligned to the IRGSP‐1.0 reference genome using HISAT2, and transcript abundance was quantified with the HTseq‐count method (D. Kim et al., 2019). Gene expression levels were normalized using DESeq2, and differentially expressed genes (DEGs) were identified based on a fold change >2 and an adjusted *p*‐value below 0.05 (Love et al., 2014). Functional enrichment analysis for gene ontology (GO) terms and Kyoto Encyclopedia of Genes and Genomes (KEGG) pathways was performed for both upregulated and downregulated DEGs using Blast2Go and the KEGG Automatic Annotation Server, respectively (Conesa et al., 2005; Moriya et al., 2007).

## 3 RESULTS

### 3.1 Characterization of HT mutant

**FIGURE 1:**
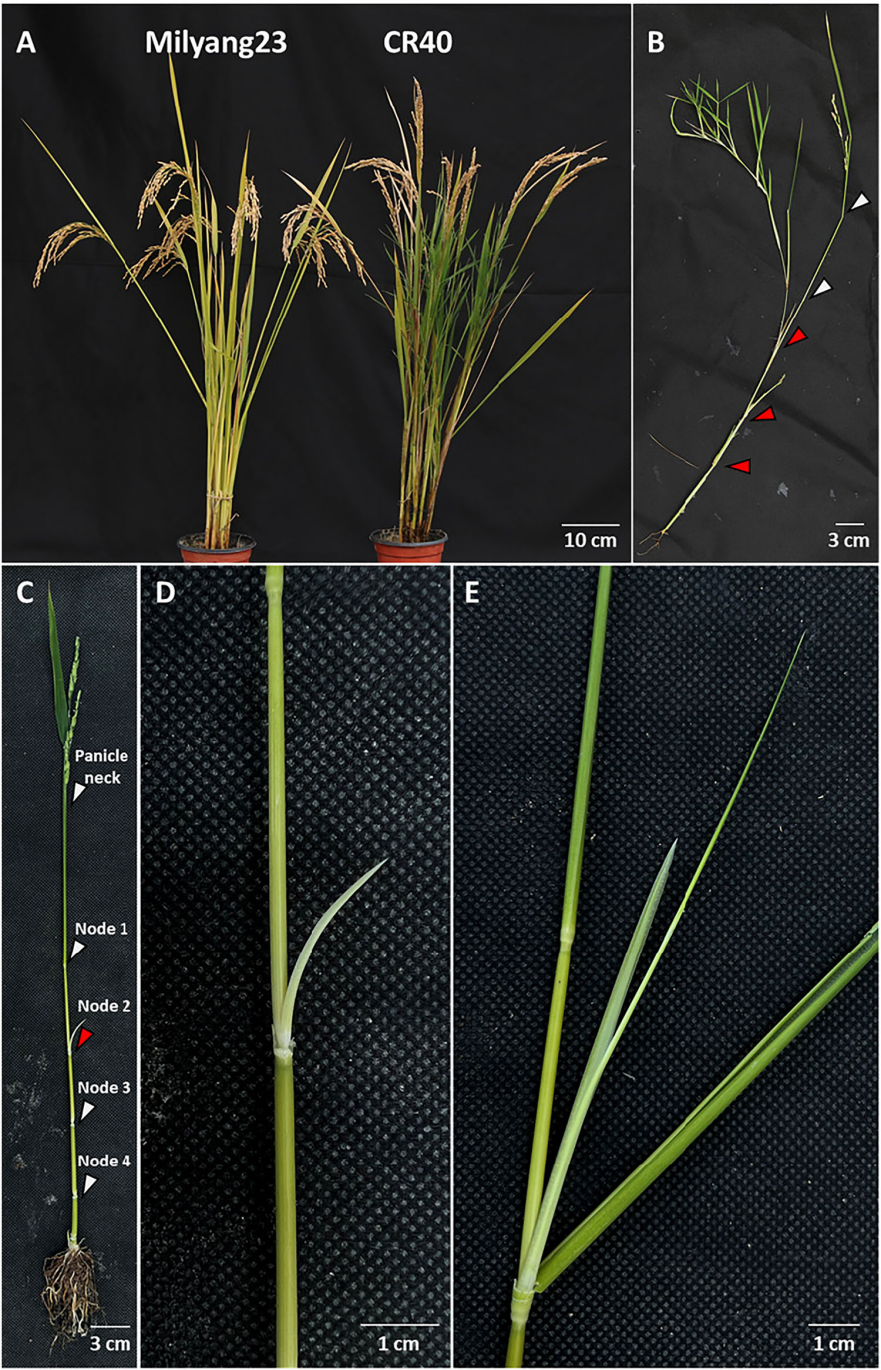
(A) Comparison of high‐tillering phenotype in Milyang23 and CR40. (B) Separated tiller of CR40 showing high tillering. Arrows indicate internodes, and red arrows indicate nodes growing high tiller. (C–E) Emergence of high tiller in CR40 after flowering stage.

An HT mutant (CR40) was identified in a population derived from a cross between the Korean Tongil‐type rice Milyang23 and weedy rice Hapcheonaengmi3 (Figure S1) (Oh et al., 2010). Interestingly, among the 68 introgression lines examined, only one line exhibited an HT phenotype. Unlike normal tillers, which emerge early in development, the HT phenotype in CR40 starts after the flowering stage (Figure 1A). In normal rice, tillers branch from nodes near the soil surface, whereas CR40 produces tillers from nodes higher on the stem (Figure 1B,C). Thin, short tillers initially develop from the second node, and this pattern repeats (Figure S4). Narrow and short leaves also emerge from these newly formed stem nodes.

When CR40 was grown during the winter season in the greenhouse, a pseudo‐vivipary phenotype was observed in the panicle, while pseudo‐vivipary was not observed under field conditions (Figure S5). To determine whether the seedlings from these HT and pseudo‐vivipary panicles could establish in soil, we separated and transplanted the stems bearing these traits into soil (Figure S6). The transplanted seedlings successfully rooted and grew normally. Moreover, this phenotype was not observed in either parental line (Milyang23 or Hapcheonaengmi3), suggesting that more than two loci may be involved in the HT phenotype, with each contributing allele derived respectively from Milyang23 and Hapcheonaengmi3.

### 3.2 Mapping of the HT loci

**FIGURE 2:**
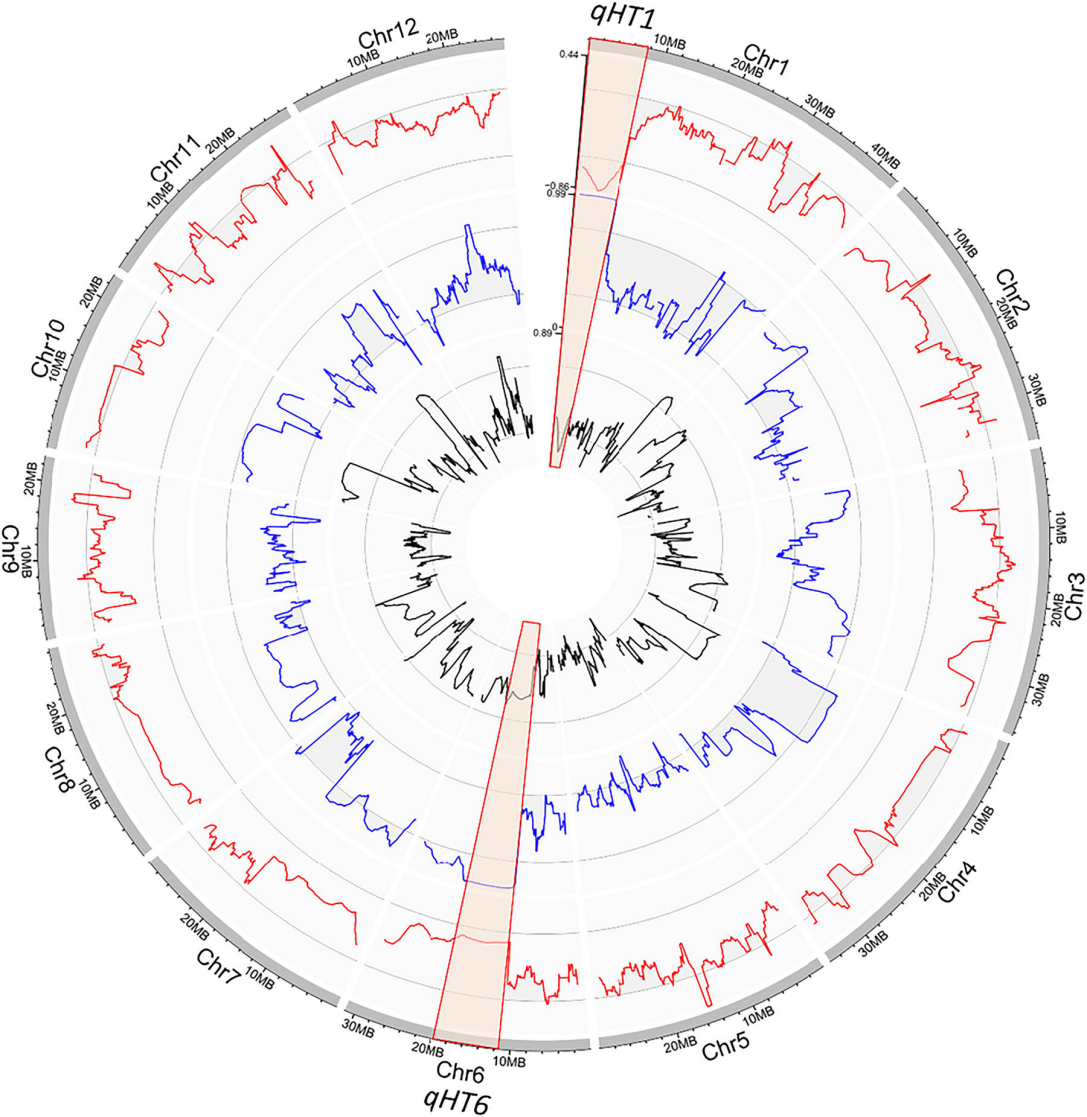
Quantitative trait locus (QTL) associated with a high‐tillering phenotype using QTL‐seq analysis. Blue and black lines indicated sliding window average of single‐nucleotide polymorphism (SNP) index of high‐tillering bulk and no‐tillering bulk, respectively. Red lines indicated delta‐SNP index. Red boxes indicated significantly associated loci based on the 95%‐confidence interval.

To identify the loci responsible for the HT trait, we crossed CR40 with Milyang23. A total of 49 lines (HT3–51) were developed, and the phenotype was evaluated from the F_4_ to F_9_ generations. HT segregated within the population, but the progeny tillering patterns were not exactly identical to those of CR40 (Figures S2 and S3). For instance, while some lines displayed a similar phenotype to CR40, others exhibited more severe, weaker, or ambiguous tillering. We used a scoring system, termed the HT score, to quantify these differences: 1 for no HT (Milyang23 type), 2 for ambiguous, and 3 for clear HT (CR40 type). Based on 6 years of evaluation data, the 49 lines were divided into two groups (Table S2; Figure S7). One group consistently showed the HT phenotype (eight lines), while the other showed no HT (12 lines). DNA from each group was bulked, and a QTL‐seq analysis was conducted (Figure 2; Table S3).

Three significant QTL regions were identified by QTL‐seq at the *p* < 0.05 significance level (Figure 2; Table 1). The first locus, designated as *qHT1* (*high‐tillering on chromosome 1*), spanned from 1 to 7.7 Mb on chromosome 1 and was significant at *p* < 0.01. The second and third loci, *qHT6.1* and *qHT6.2*, were located at 11.1–19.9 Mb and 24.3–27 Mb on chromosome 6, respectively, and were significant at *p* < 0.05. At all three loci, Hapcheonaengmi3 alleles were associated with the induction of the HT phenotype. On chromosome 6, the delta‐SNP index exhibited a continuous elevation from 11.1 to 27 Mb; however, it decreased below the significance threshold in the 20–24 Mb interval. This discontinuity raises the question of whether the chromosome 6 signal represents a single broad QTL or two distinct loci.

**TABLE 1:** Quantitative trait loci (QTLs) identified for the high‐tillering trait using QTL‐seq analysis.

QTL	Chromosome	Start (bp)	End (bp)	Size (Mb)	*p*‐value	Allele effect
*qHT1*	1	1	7,700,000	7.7	<0.01	HP3^a^
*qHT6.1*	6	11,100,000	19,900,000	8.8	<0.05	HP3
*qHT6.2*	6	24,300,000	27,000,000	2.7	<0.05	HP3

^a^ HP3 indicates Hapcheonaengmi3.

### 3.3 Confirmation and fine‐mapping of the HT QTL

To verify the QTLs identified by the QTL‐seq analysis, we conducted linkage analysis for the *qHT1* and *qHT6* regions. A new F_2_ population was developed from a cross between Milyang23 and HT35, which exhibits a more severe HT phenotype than CR40 (Figure S3). In addition, HT35 is a progeny line derived from the CR40 × Milyang23 population, and therefore its genome composition is more similar to that of Milyang23 than CR40. Based on this, we expected that the HT35 × Milyang23 cross would reduce background noise and produce more precise QTL results. A total of 204 F_2_ plants were generated and genotyped for the *qHT1* and *qHT6* QTL regions using InDel markers (Tables S1 and S4). For HT phenotyping, we used a numerical score based on the degree of HT, ranging from 1 (no HT, Milyang23 type) to 9 (severe HT, HT35 type).

**FIGURE 3:**
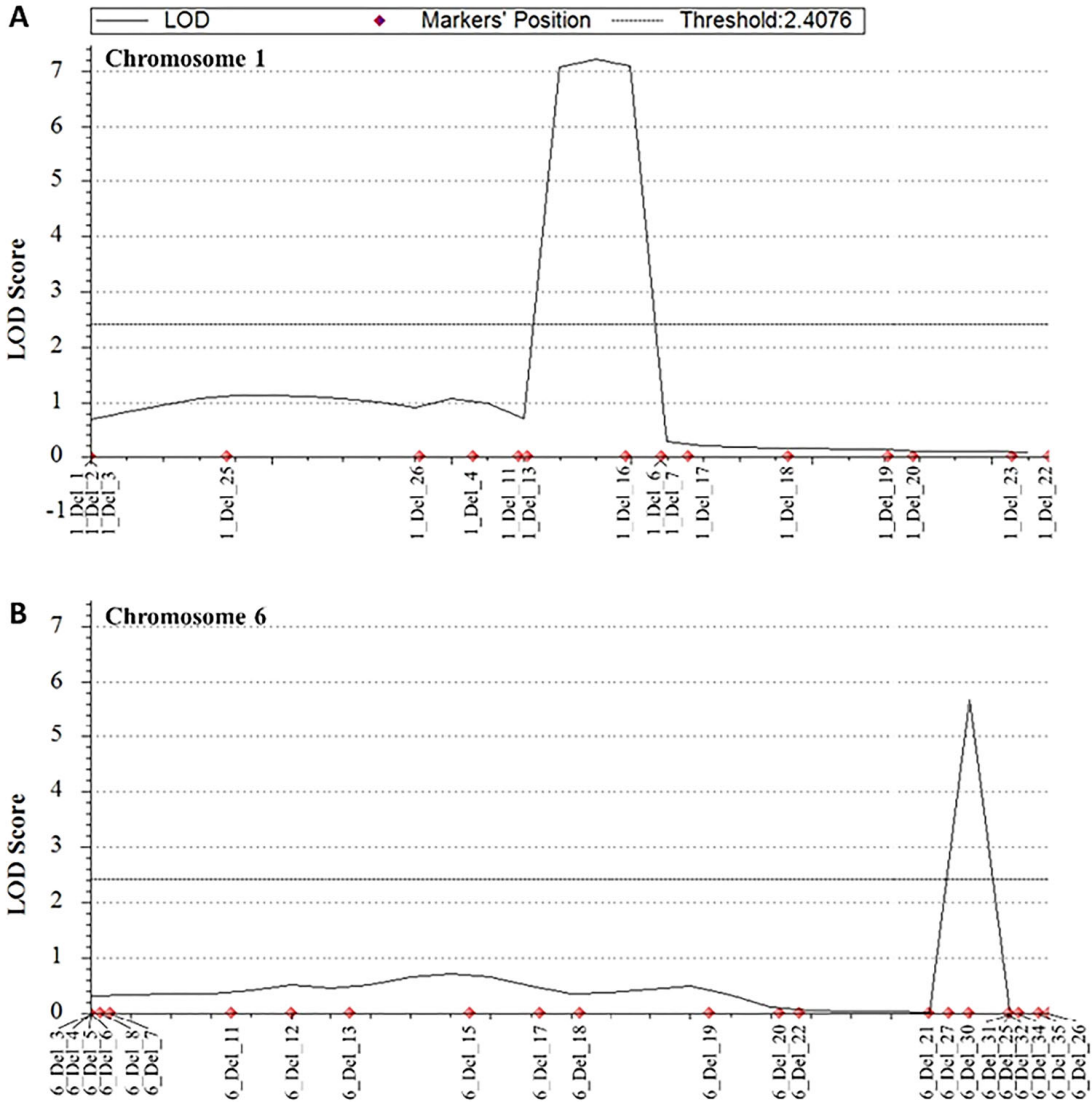
Validation of quantitative trait locus (QTL) for high‐tillering traits on (A) chromosomes 1 and (B) chromosome 6 using an F_2_ population. The logarithm of odds (LOD) score was calculated using a permutation test with 1000 iterations at a 0.05 significance level. QTL analysis was performed using the ICIM‐ADD method in QTL IciMapping version 4.2. The black line represents the LOD score across the chromosome, while the red diamond markers indicate the positions of InDel markers used for the analysis. The dashed horizontal line represents the significance threshold (LOD = 2.4076).

QTL analysis in the F_2_ population narrowed *qHT1* to a 418 kb interval (3,184,505–3,603,180 bp) between the 1_InDel_13 and 1_InDel_16 markers (Figure 3A). The LOD value for *qHT1* was 7.2, explaining 15.2% of the phenotypic variation (Table 2). Meanwhile, *qHT6* was mapped to a 106 kb region (27,093,056–27,199,417 bp) between the 6_InDel_30 and 6_InDel_31 markers, with an LOD score of 5.7, explaining 11.7% of the phenotypic variation (Figure 3B; Table 2). These findings confirm that the QTL‐seq results are consistent with the F_2_ QTL analysis, indicating that the two Hapcheonaengmi3 alleles at *qHT1* and *qHT6* are responsible for the HT phenotype and two loci of Hapcheonaengmi3 alleles induced HT phenotype in the Milyang23 genetic background. Furthermore, based on the LOD and *R* ^2^ values, the significance of *qHT1* was higher than *qHT6* in the linkage analysis. This result aligns with the QTL‐seq analysis, where *qHT1* was significant at the *p* < 0.01 level, while *qHT6* was significant at the *p* < 0.05 level. Additionally, the analysis suggests that the *qHT6* locus identified through QTL‐seq represents a single QTL rather than two separate loci.

**TABLE 2:** Quantitative trait locus (QTL) confirmation and phenotypic variation explained in the F_2_ population.

Trait	QTL	Chr.	Left marker	Right marker	LOD	PVE (%)	Add	Dom
HT	*qHT1*	1	1_Del_13	1_Del_16	7.23	15.22	1.63	0.60
	*qHT6*	6	6_Del_30	6_Del_31	5.67	11.70	1.04	1.25

Abbreviations: Add, additive effect; Chr., chromosome; Dom, dominance effect; HT, high‐tillering phenotype; LOD, logarithm of odds; PVE, phenotypic variance explained.

### 3.4 Candidate gene analysis of *qHT1* and *qHT6*

**FIGURE 4:**
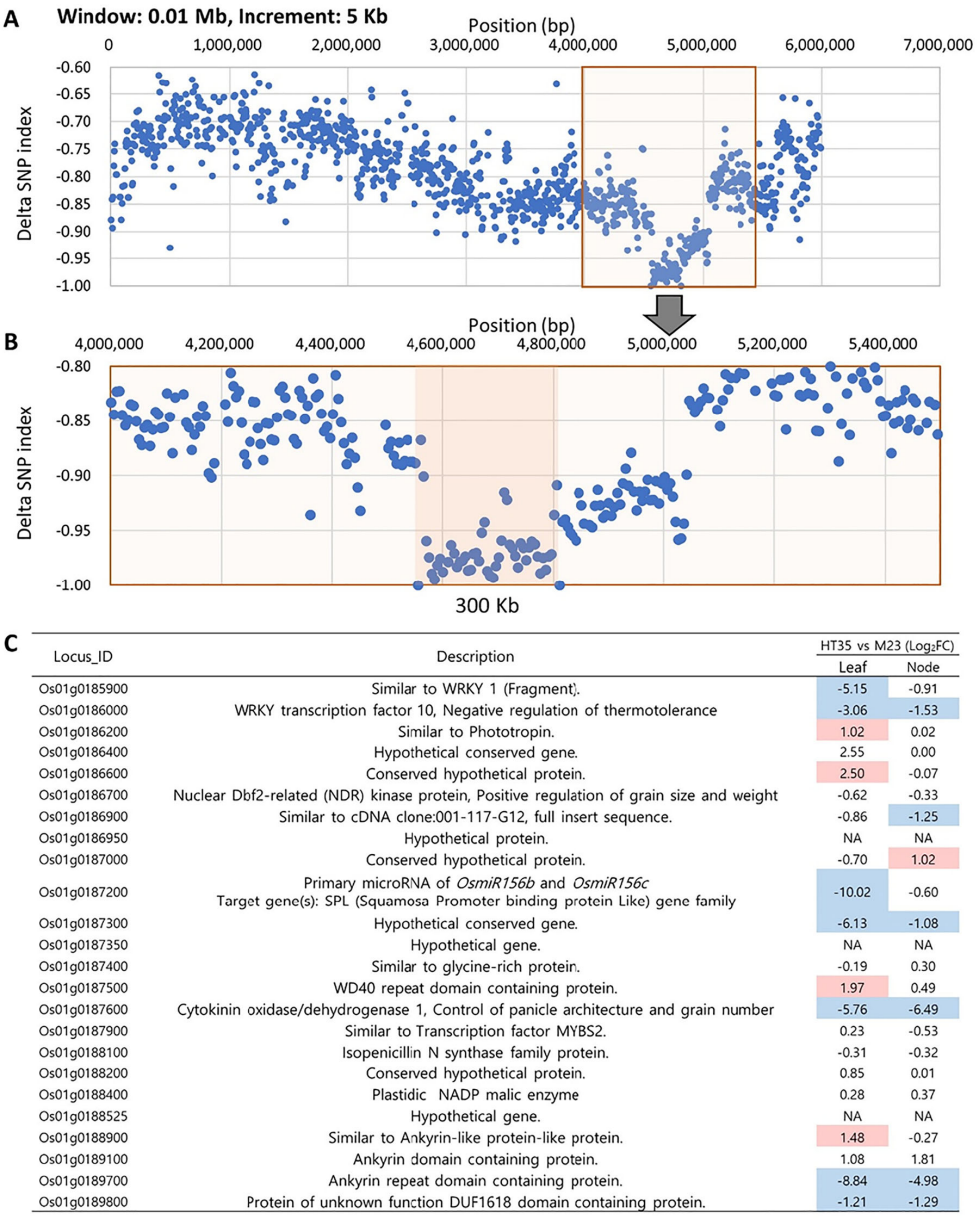
Identification of a candidate gene in the *qHT1* region using a significant delta single‐nucleotide polymorphism (SNP) index at *p* < 0.01. (A) Sliding window analysis with a 0.01 Mb window size and a 5 kb increment. The highlighted region indicates a potential candidate region for the target trait. (B) A magnified view of the highlighted region in (A), showing the detailed SNP distribution within the candidate interval. (C) Gene list of the 300 kb candidate region (*qHT1.2*) with Log_2_ fold‐change values generated from RNA‐seq analysis. Colored columns indicate significantly differentially expressed genes (DEGs), with red and blue representing upregulated and downregulated genes, respectively. Expression comparison was conducted between HT35 and Milyang23 (M23). Samples were taken from the leaf of the high‐tillering HT35, the flag leaf of M23, and the node of both HT35 and M23.

The *qHT1* and *qHT6* regions were mapped to 418 and 106 kb, respectively, based on linkage analysis. To further explore the locus associated with HT within the QTL‐seq results, additional refinement based on SNP index data were performed (Figure 4). Specifically, for the *qHT1* region, the mean delta SNP indices within a 4 Mb region identified by the initial sliding window analysis were lower than those in surrounding regions (Figure 2), suggesting the presence of a candidate interval. Therefore, we applied sliding window analyses using statistically significant sequence variants (SNPs and InDels) at the *p* < 0.01 level. To examine local variation patterns within this broader region, the window size and increment were gradually reduced relative to the initial analysis (Figure S8). The use of different window parameters resulted in variable mapping resolutions, as expected for bin‐based SNP index analyses. Using a window size of 0.01 Mb with a 5 kb increment (Figure 4A), we identified a 300 kb interval (4,556,151–4,811,151 bp) that showed consistently strong delta SNP index signals (Figure 4B). This interval was located approximately 1.0–1.6 Mb away from the region identified through linkage analysis. Rather than definitively concluding the presence of two independent loci, we interpret these regions as adjacent candidate intervals derived from distinct analytical approaches, recombination‐based linkage mapping and physical position‐based SNP index analysis. Accordingly, for clarity in subsequent candidate gene analysis, these intervals were designated *qHT1.1* (linkage‐derived) and *qHT1.2* (sliding window‐derived), pending further validation.

To identify candidate genes within these QTLs, annotated genes were downloaded from the RAP‐DB database (https://rapdb.dna.affrc.go.jp/). A total of 64 genes were annotated in the *qHT1.1* region, with 43 genes having specific descriptions, including leucine‐rich repeat proteins, transcription factors, and zinc finger proteins (Table S5). In the *qHT1.2* region, a total of 24 genes were identified, including transcription factors, primary microRNA, and cytokinin oxidases (Figure 4C). For the *qHT6*, nine genes were annotated, with five genes having specific descriptions (Table S6). SQUAMOSA PROMOTER‐BINDING PROTEIN, intron maturase, actin filament bundling protein, DUF231, and glutaredoxin genes were included in *qHT6*.

In the *qHT6* region, nine candidate genes were identified, and one of them encodes an SPL transcription factor, *OsSPL10* (Os06g0659100), which has been reported to play a role in seedling‐stage salt tolerance and trichome formation (Lan et al., 2019). In addition, the primary microRNA *OsmiR156b* and *OsmiR156c* genes (hereafter *pri‐miR156b/c*; Os01g0187200) were identified within the *qHT1.2* region. *OsmiR156* is known to be strongly associated with the transcriptional regulation of *SPL* genes, and previous studies have reported that the *SPL–miR156* regulatory module is responsible for HT phenotype (Hayashi‐Tsugane et al., 2015). Therefore, Os06g0659100 and Os01g0187200 were prioritized as candidate genes potentially contributing to the HT phenotype, pending functional validation.

**FIGURE 5:**
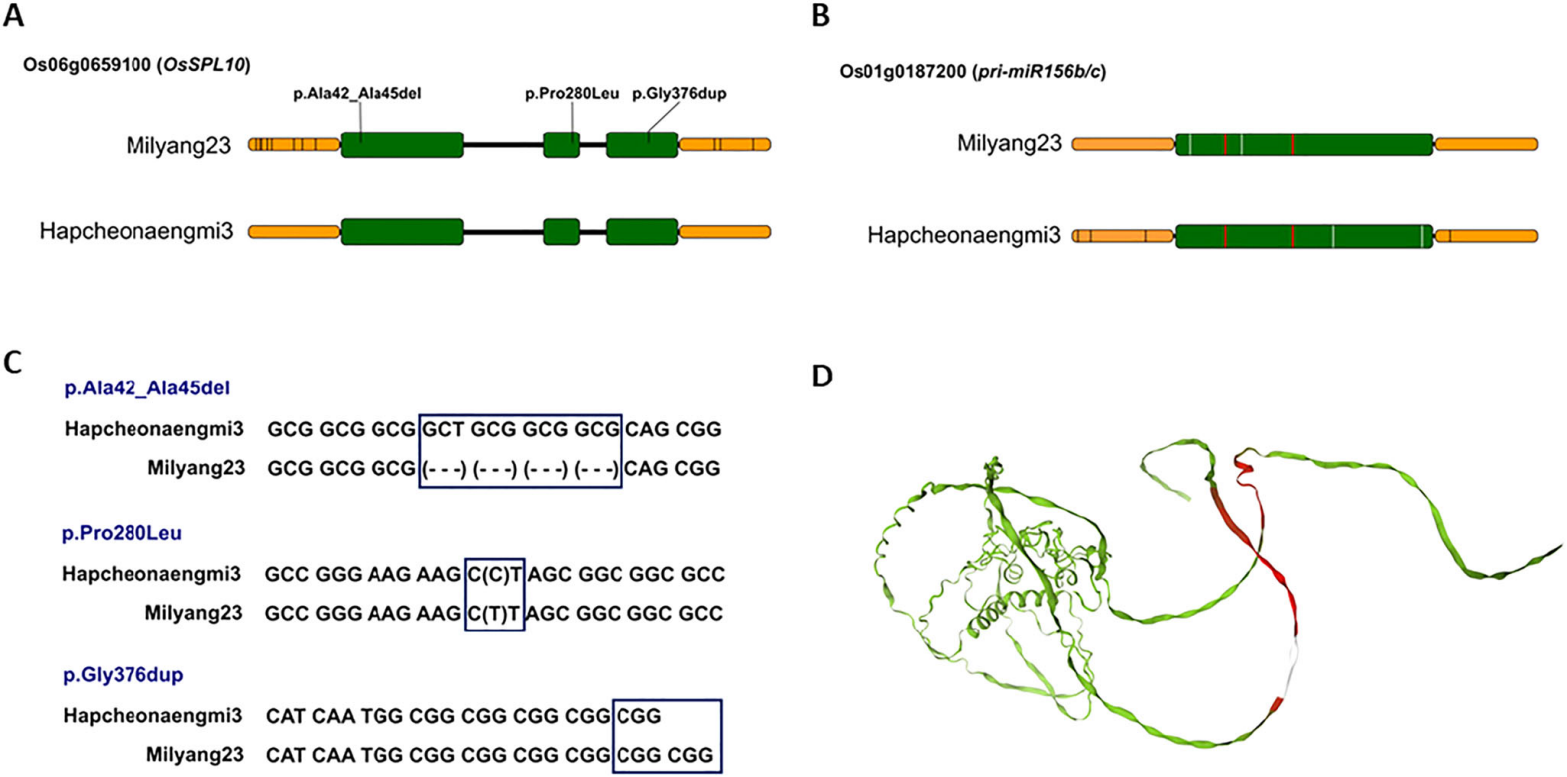
Sequence variations in *OsSPL10* and primary microRNA156 genes between two parental lines, Milyang23 and Hapcheonaengmi3. (A) Schematic comparison of the *OsSPL10* (Os06g0659100) gene structure. Promoter and exon regions are represented by yellow and green boxes, respectively. Vertical lines (black) indicate variations within the promoter region. Genetic variations within the coding sequence of Milyang23 are labeled with their predicted amino acid changes. (B) Gene structure comparison of the *primary microRNA156* gene (Os01g0187200) locus. Vertical lines (red) indicate mature miRNA sequence (miRNA156b, c). (C) Nucleotide sequence alignments for the three major variations identified in the *OsSPL10* exons of Milyang23 compared to Hapcheonaengmi3. (D) Superimposition of the predicted 3D protein structures of *OsSPL10* from Milyang23 and Hapcheonaengmi3, modeled using AlphaFold2. Regions with significant structural differences between the two models are highlighted in red. The loop region corresponding to the p.Ala42_Ala45del deletion in Milyang23 is indicated in white.

To investigate the genetic basis of the phenotypic differences, we analyzed the genomic sequences of the candidate genes (Figure 5). Sequence alignment of *OsSPL10* revealed distinct variations between the two parental lines. While no distinguishable variations were detected in Hapcheonaengmi3 when compared to the rice reference genome, Milyang23 harbored specific non‐synonymous mutations within the coding region, including a deletion (p.Ala42_Ala45del), a substitution (p.Pro280Leu), and a duplication (p.Gly376dup) (Figure 5A,C). Furthermore, multiple variations were observed in the promoter region of *OsSPL10* in Milyang23. To assess the potential impact of these coding sequence variations on protein function, we predicted the 3D structures of *OsSPL10*. Superimposition of the protein models suggested potential structural deviations associated with the identified mutations. However, the functional consequences of these predicted changes remain to be experimentally validated (Figure 5D). In the case of *pri‐miR156b/c*, variations were confined to the promoter or non‐mature sequences, with no changes observed within the mature miRNA sequence that would alter its target specificity (Figure 5B). This suggests that the functional divergence of *pri‐miR156b/c* between the two varieties is likely driven by transcriptional regulation mediated by promoter polymorphisms rather than structural changes in the miRNA itself. Importantly, the HT phenotype was not observed in either parental line, suggesting that these sequence variations alone are insufficient to explain the phenotype. Instead, HT likely results from an interaction between alleles at *qHT1* and *qHT6* within a specific genetic background. Therefore, rather than attributing HT to individual coding mutations, we propose that allelic interactions affecting regulatory balance within the miR156–SPL module underlie the emergent phenotype.

**FIGURE 6:**
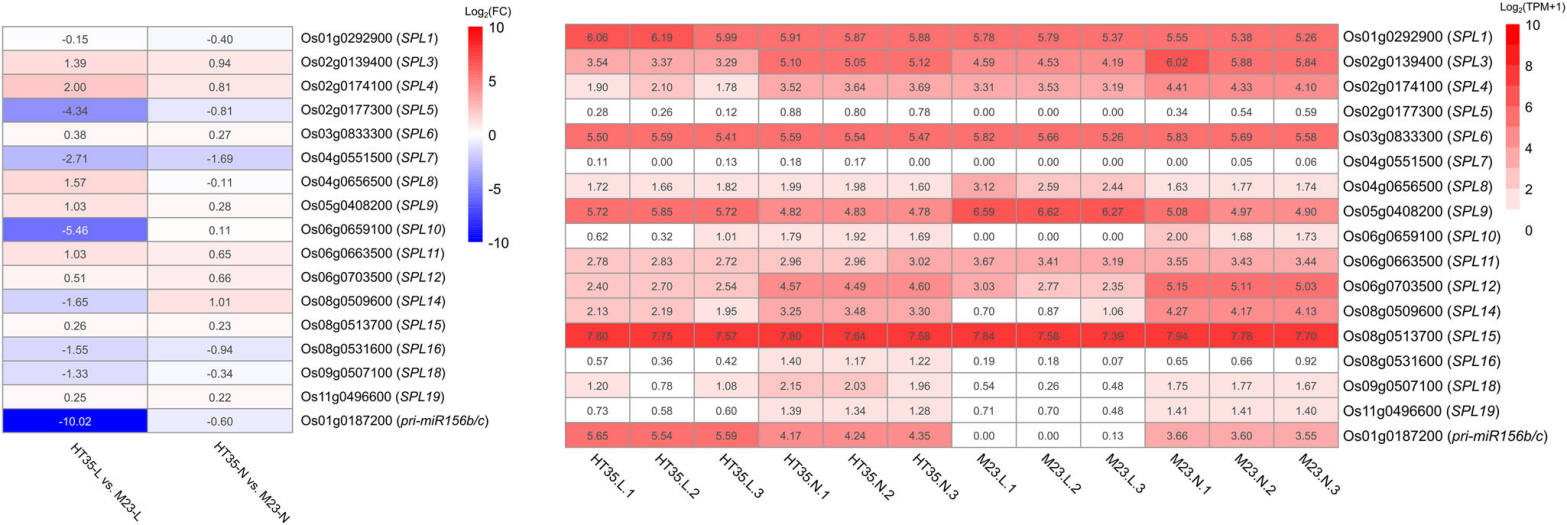
Expression profile analysis of *OsSPL* and *pri‐miR156b/c* genes in HT35 and Milyang23 (M23) varieties. (A) Heatmap representing the Log_2_ fold‐change values of *OsSPL* family genes and *pri‐miR156b/c*. Log_2_ fold‐change values were calculated as log_2_(M23/HT35), where negative values indicate higher expression in HT35 and positive values indicate higher expression in M23. C1 represents the leaf comparison (HT35‐L vs. M23‐L), and C6 represents the node comparison (HT35‐N vs. M23‐N). (B) Heatmap showing the normalized expression values (log_2_(TPM+1)) for each gene across three biological replicates. Samples correspond to leaf (L) and node (N) tissues from HT35 and M23 varieties. The color scale from white to red indicates low to high expression levels.

To verify whether these promoter and structural variations lead to transcriptional differences, we analyzed the expression profiles of the *OsSPL* gene family and the *pri‐miR156b/c* in leaf and node tissues of Milyang23 and HT35 using RNA‐seq (Figure 6). Four types of samples, which are the HT leaf of HT35 (HT35‐L), the tiller‐emerging second node of HT35 (HT35‐N), the flag leaf of Milyang23 (M23‐L), and the second node of Milyang23 (M23‐N), were subjected to RNA‐seq with three biological replicates. The transcriptome data revealed distinct expression patterns between the HT‐HT35 and the control Milyang23. Notably, *pri‐miR156b/c* exhibited a drastic difference in expression, with a log_2_FoldChange (log_2_FC) of −10.02 in leaf tissue (Figure 6A), indicating substantially higher expression in Hapcheonaengmi3 compared to Milyang23. Similarly, *OsSPL10* showed significant differential expression (log_2_FC −5.46), being markedly more abundant in HT35. Beyond these specific candidates, the heatmap reveals a broader restructuring of the *SPL* gene expression landscape (Figure 6B). While the specific interaction dynamics between the two candidate genes require further elucidation, these results suggest that the introgression of *OsSPL10* and *pri‐miR156b/c* alleles from Hapcheonaengmi3 into the Milyang23 genetic background triggers a systemic shift in the expression of the *SPL* gene family. We propose that this global modulation of the *SPL*‐*miR156* regulatory network, resulting from these introgressed alleles, is the primary driver underlying the HT phenotype.

### 3.5 Comprehensive transcriptome analysis for the HT line

**FIGURE 7:**
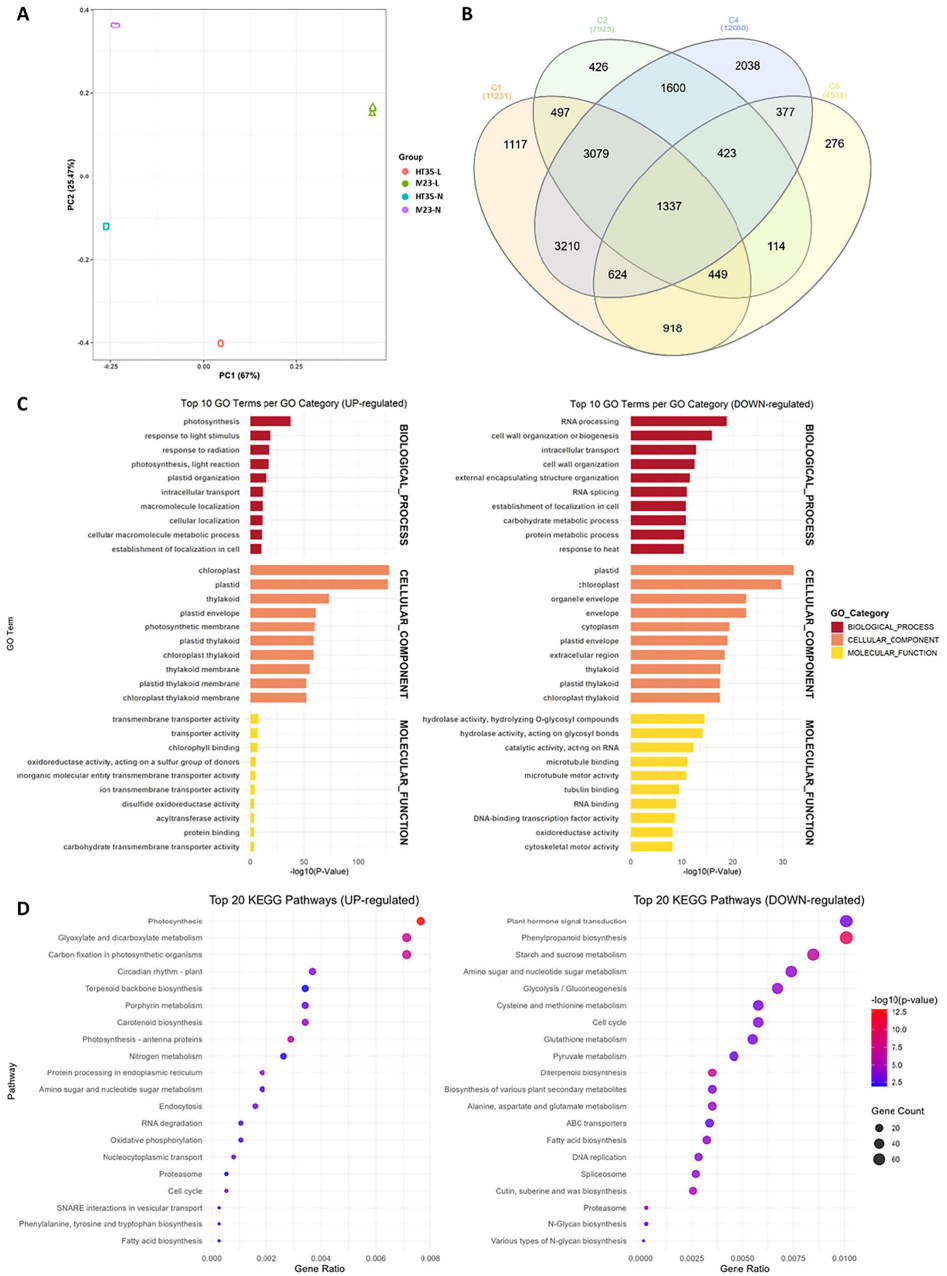
Transcriptome analysis using leaf and node tissues of HT35 and M23. To identify differentially expressed genes (DEGs), pairwise comparisons were conducted between the following groups: HT35 leaf versus M23 leaf (C1), HT35 leaf versus HT35 2nd node (C2), HT35 leaf versus M23 2nd node (C3), M23 leaf versus M23 2nd node (C4), HT35 2nd node versus M23 leaf (C5), and HT35 2nd node versus M23 2nd node (C6). (A) Principal component analysis (PCA) of transcriptome data from the high‐tillering leaf of HT35 (HT35‐L), the 2nd node from which tillers emerge in HT35 (HT35‐N), the flag leaf of Milyang23 (M23‐L), and the 2nd node of Milyang23 (M23‐N). (B) Venn diagram of DEGs from C1, C2, C4, and C6. (C) Gene ontology (GO) analysis and (D) Kyoto Encyclopedia of Genes and Genomes (KEGG) pathway analysis of DEGs from C1.

To further elucidate the broader transcriptional landscape associated with HT development, we performed a comprehensive transcriptome analysis using the RNA‐seq dataset generated from leaf and node tissues of HT35 and Milyang23. Principal component analysis confirmed clear separation of the samples according to genotype and tissue type, with tight clustering among biological replicates (Figure 7A). This indicates high data consistency and highlights genotype‐specific transcriptional signatures underlying the HT trait.

To identify DEGs, pairwise comparisons were conducted: HT35‐L versus M23‐L (C1), HT35‐L versus HT35‐N (C2), HT35‐L versus M23‐N (C3), M23‐L versus M23‐N (C4), HT35‐N versus M23‐L (C5), and HT35‐N versus M23‐N (C6) (Table 3). From comparisons C1–C6, a total of 11,231, 7925, 9857, 12,688, 13,677, and 4518 DEGs, respectively, were identified. Among these, we prioritized C1, C2, C4, and C6, which compare either the same genotype or the same tissue type. A Venn diagram was generated to illustrate the overlap of DEGs across these comparisons (Figure 7B). Notably, in comparisons C1 and C6, which involved the same tissue, 2255 DEGs were shared, while 1117 and 276 DEGs were uniquely identified in C1 and C6, respectively.

**TABLE 3:** Summary of differentially expressed genes in pairwise transcriptome comparisons.

Comparison	A vs. B	UP (A < B)	DOWN(A > B)	Total
C1	HT35‐L vs. M23‐L	3797	7434	11,231
C2	HT35‐L vs. HT35‐N	4427	3498	7925
C3	HT35‐L vs. M23‐N	4692	5165	9857
C4	M23‐L vs. M23‐N	7593	5095	12,688
C5	HT35‐N vs. M23‐L	4973	8704	13,677
C6	HT35‐N vs. M23‐N	1524	2994	4518

*Note*: HT35‐L, M23‐L, HT35‐N, and M23‐N indicate high‐tillering leaf tissue from HT35, flag leaf from Milyang23, node tissue from HT35, and node tissue from Milyang23, respectively.

To gain further insight into the biological functions associated with DEGs from C1, GO analysis was performed (Figure 7C). In the biological process, photosynthesis, response to light stimulus, and photomorphogenesis were significantly enriched in the up‐regulated DEGs, indicating alterations in photosynthetic activity and light perception. In contrast, down‐regulated genes were primarily associated with RNA processing, cell wall organization, intracellular transport, and protein localization, suggesting shifts in cellular activities related to structural organization and transcriptional regulation. Within the cellular component, genes involved in the chloroplast, plastid, and thylakoid were up‐regulated, whereas apoplast, cytoplasmic vesicles, and protein complexes were down‐regulated, highlighting potential changes in cellular transport mechanisms and structural organization between these samples. For the molecular function, up‐regulated DEGs were predominantly associated with chlorophyll binding, transporter activity, and ATPase activity, all of which are crucial for energy production and metabolite transport. Conversely, down‐regulated genes were enriched in DNA‐binding transcription factor activity, metal ion binding, and catalytic activity, suggesting alterations in regulatory and enzymatic functions.

KEGG pathway analysis further revealed that photosynthesis, glyoxylate and dicarboxylate metabolism, and carbon fixation in photosynthetic organisms were enriched among upregulated DEGs, whereas plant hormone signal transduction, phenylpropanoid biosynthesis, and starch and sucrose metabolism were enriched among downregulated DEGs (Figure 7D). Overall, these findings indicate that the HT tissue of HT35 and the flag leaf of M23 exhibit distinct expression patterns not only in photosynthetic capacity and energy production but also in structural maintenance and transcriptional regulation. Furthermore, plant hormone signaling pathways may play an important role in the development of HT.

### 3.6 Plant hormone‐related gene expression analysis in C1 and C6

**FIGURE 8:**
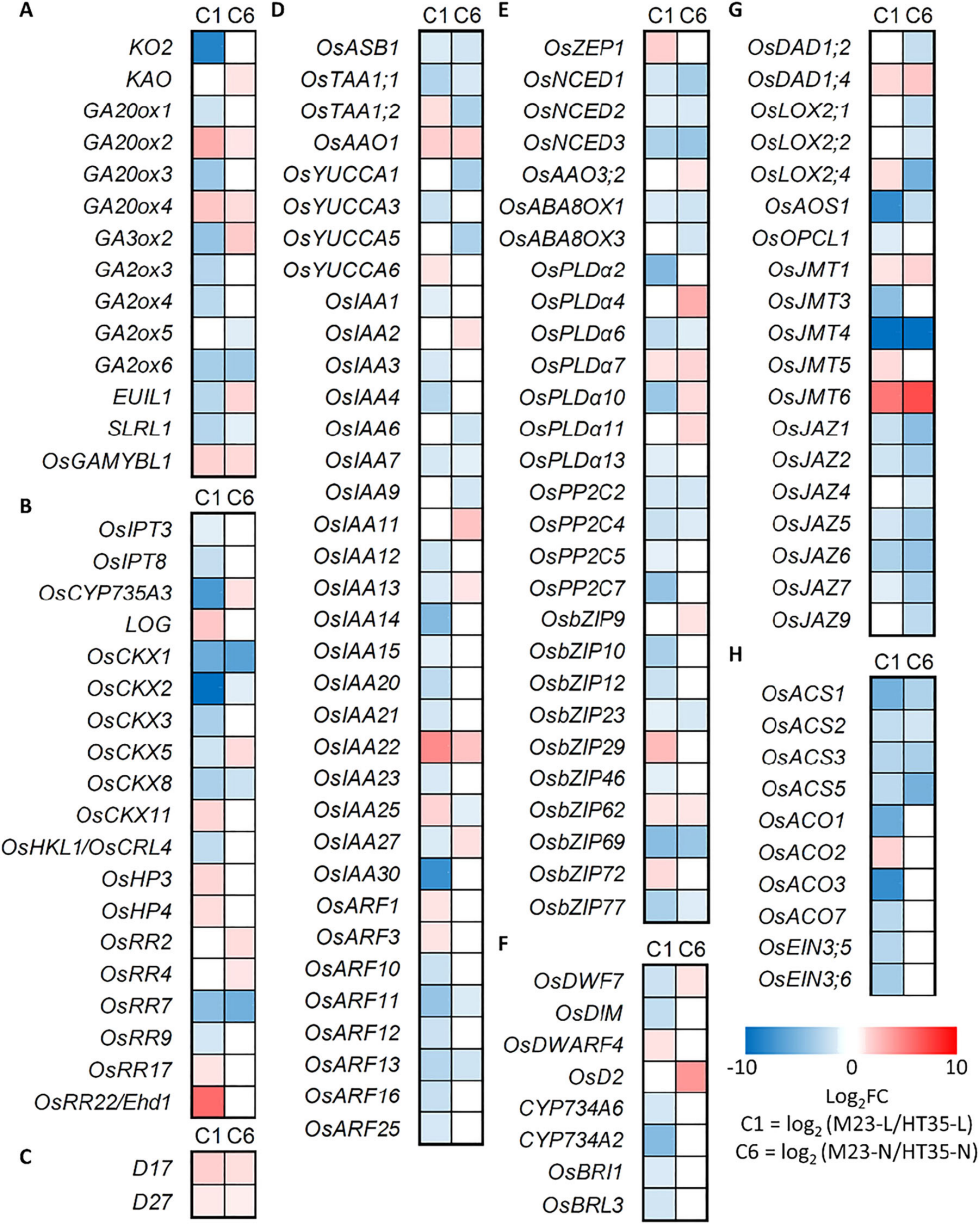
Transcriptome profiling of plant hormone‐related genes using DEGs from HT35‐L versus M23‐L (C1) and HT35‐N versus M23‐N (C6). Differentially expressed genes (DEGs) related to plant hormones: (A) gibberellin, (B) cytokinin, (C) strigolactone, (D) auxin, (E) abscisic acid, (F) brassinosteroid, (G) jasmonic acid, and (H) ethylene. Blue and red indicate higher gene expression in HT35 and M23, respectively.

The KEGG analysis result showed that DEGs from C1 were associated with plant hormone signal transduction. To better understand the association between HT and plant hormone pathways, plant hormone‐related genes were examined (Figure 8). A suite of genes that are involved in diverse plant hormone pathways was identified among the DEGs from C1 and C6. Several genes were involved in GA metabolism, including *KO2, KAO, GA20ox, GA3ox, GA2ox*, and *EUI1*. These genes displayed large negative log_2_FoldChange (log_2_FC) values, suggesting higher expression in HT35 (Figure 8A). In addition, the GA‐related regulator *SLR1* and the GA‐responsive gene *OsGAMYBL1* were both significantly altered in C1 and C6. Similarly, several cytokinin metabolism genes, such as members of the *OsCKX* (cytokinin oxidase/dehydrogenase) family, were differentially expressed, suggesting that cytokinin homeostasis could be critical for the HT phenotype (Figure 8B). In addition, several genes involved in strigolactone biosynthesis and signaling, including *D17* and *D27*, were also differentially expressed (Figure 8C). These genes encode key enzymes required for early steps of SL biosynthesis and showed positive log_2_FC values, indicating higher expression in Milyang23. Some *OsCKXs* showed large differences in gene expression level, and particularly, *OsCKX2*, previously reported as a major QTL for grain number (*gn1a*), exhibited a −11 log_2_FC value (Ashikari et al., 2005). Many *OsIAA* family members, which are auxin‐related genes, showed substantial negative log_2_FC values (Figure 8D). Among the *IAA* genes, *OsIAA30* which is a previously reported gene controlling many agronomic traits, exhibited −7 log_2_FC value (He et al., 2022). Beyond GA, auxin, and cytokinin, genes associated with brassinosteroid, abscisic acid, and jasmonic acid pathways also displayed negative log_2_FC values, implying upregulation in HT35 and highlighting complex hormonal crosstalk in tiller initiation and growth (Figure 8E–G). Moreover, genes linked to ethylene were differentially expressed (Figure 8H). Taken together, these results suggest that plant hormone‐related DEGs in HT35 demonstrate extensive reprogramming of hormone biosynthesis and signaling networks, and plant hormone would be a key driver of the increased tiller production in HT35.

## 4 DISCUSSION

### 4.1 HT phenotype arises from novel allelic interactions

In this study, we identified a novel HT phenotype in the progeny of a cross between a Korean weedy rice (Hapcheonaengmi3) and a Tongil‐type rice (Milyang23). Neither parental line exhibited the HT phenotype, indicating that the unique allelic combination from both parents drives the emergence of the HT phenotype. Similar observations have been reported in other hybrid weakness traits, where interacting loci from divergent lines produce phenotypes absent in the respective parents (Chen et al., 2014; S. H. Kim et al., 2022). Our QTL mapping results showed that two Hapcheonaengmi3 loci, *qHT1* and *qHT6*, induce the HT phenotype in the Milyang23 genetic background. This multi‐locus regulation of the HT phenotype could be a reason why this phenotype and its genetic basis have not been reported previously. Although QTL‐seq initially suggested that the region on chromosome 6 might represent two sub‐loci, subsequent genotyping and linkage mapping supported a single QTL of a relatively large genomic region.

A critical aspect of this study is that the HT phenotype was not present in either parental line but emerged only after combining alleles from Hapcheonaengmi3 and Milyang23. This indicates that HT is not caused by a simple gain‐ or loss‐of‐function mutation in a single gene but rather by a previously hidden allelic interaction between divergent genetic backgrounds. Such emergent phenotypes highlight the importance of epistatic regulation and cryptic variation in shaping complex plant architecture. Therefore, the novelty of this study lies in demonstrating how naturally occurring alleles derived from weedy rice can reconfigure conserved developmental modules and generate a previously unobserved architectural phenotype.

Importantly, the genetic architecture underlying HT appears to be polygenic, involving at least two independent loci (*qHT1* and *qHT6*) that jointly contribute to the phenotype. This multi‐locus control likely explains both the rarity of the HT phenotype among introgression lines and the difficulty in pinpointing a single causal gene. While this complexity represents a limitation in terms of definitive gene identification, it simultaneously underscores a key contribution of our study: the dissection and validation of multiple interacting QTLs that collectively drive a novel architectural outcome. The identification of these loci provides a foundational framework for future fine‐mapping and functional validation aimed at resolving the precise genetic determinants of HT.

### 4.2 Dissection of *qHT1* and *qHT6* loci through multi‐population mapping

The *qHT1* locus was narrowed down with a sliding window approach and linkage analysis using two independent populations. Sliding window has been facilitated for bioinformatics era to study properties of chromosome sequences (Zhu et al., 2020). QTL‐seq analysis also employed a sliding window approach to identify associated loci to the trait (Takagi et al., 2013). However, previous studies have not reported conducting fine‐mapping by progressively modifying the parameters of sliding window analysis. In our sliding window analysis, window size and increment were progressively decreased, resulting in a high‐resolution mapping interval of approximately 300 kb (Figure S8). This stepwise refinement method represents an extension of the conventional QTL‐seq pipeline, which typically applies a fixed window size. To our knowledge, such iterative narrowing of the search window has not been widely utilized in fine‐mapping, making it a potentially powerful strategy for pinpointing precise genomic regions associated with complex traits. This strategy could serve as a valuable addition to the QTL‐seq methodology, potentially making it more powerful for fine‐mapping when coverage and population sizes permit.

However, the *qHT1* locus was fine‐mapped into two separate regions, *qHT1.1* and *qHT1.2*, using the sliding window approach and linkage analysis, respectively, with an interval of approximately 1.0–1.6 Mb between them. Although the two fine‐mapped loci were close enough to validate the QTL, their separation made it difficult to identify the causal gene responsible for the HT phenotype. Similar results were observed from previous QTL‐seq study (Takagi et al., 2013). A total of 241 recombinant inbred lines were used to detect QTLs for blast resistance, derived from a cross between Nortai and Hitomebore. Using the QTL‐seq method and linkage analysis, QTL mapping was conducted, revealing that the peak delta SNP index from QTL‐seq and the peak LOD score from linkage analysis were slightly different. These discrepancies between QTL‐seq and linkage analysis were likely due to differences in analytical methodologies. The QTL‐seq approach utilized physical positions and SNP indices from sequencing reads, whereas the linkage map relied on genetic distances and recombination frequencies to determine the relative positions of loci. To precisely identify the locus responsible for HT, additional backcrossing and substitution mapping will be required. In particular, the development of near‐isogenic lines carrying *qHT1* and *qHT6* alleles in a uniform genetic background will be essential to dissect their individual and epistatic effects.

### 4.3 Candidate genes and the *SPL–miR156* regulatory module

In the *qHT6* region (Chr. 6: 27,093,056–27,199,417), nine candidate genes were identified (Table S6). One of these candidate genes encodes an *SPL* transcription factor, OsSPL10 (Os06g0659100), which plays a role in salt tolerance at the seedling stage and trichome formation (Lan et al., 2019). Additionally, another SPL family gene, *OsSPL11*, is located on chromosome 6 at the physical position 27,387,616–27,391,161, 188 kb away from *qHT6*. Although our mapping result delimited qHT6 between the marker interval 6_Del_30 and 6_Del_31, OsSPL11, located approximately 188 kb outside this region, could still be considered a candidate gene for the HT phenotype due to its known regulation by *miR156* and its established role in axillary meristem development (Xie et al., 2006). Interestingly, in the *qHT1.2* region, Os01g0187200 encodes the primary microRNA of *OsmiR156b* and *OsmiR156c*, and its expression in HT35 was 10‐fold higher than that in Milyang23 in leaf samples (Figure 6). miRNAs play important roles in various stages of plant development by targeting complementary mRNAs and suppressing their gene expression and translation (Xie et al., 2006). Overexpression of *OsmiR156b* caused dramatic morphological changes, including reduced plant height and an increased number of tillers (Xie et al., 2006). Additionally, Hayashi‐Tsugane et al. (2015) reported that increased expression of miR156d is associated with bushy and dwarf tiller phenotypes in rice, and this morphology resembles the HT phenotype observed in CR40. Therefore, the *miR156–SPL* module could represent a candidate regulatory module underlying the HT phenotype. In our study, expression profiling of the *SPL* gene family revealed substantial expression changes in both SPL genes and *pri‐miR156b/c* when comparing leaf and node tissues of HT35 and Milyang23, supporting the idea that altered regulation of this module plays a central role in HT development. Together, these findings suggest that coordinated dysregulation of the *miR156–SPL* regulatory cascade may drive enhanced axillary meristem activity and excessive tiller formation in the HT35. Variations and interactions of primary microRNA and *SPL* genes between Milyang23 and Hapcheonaengmi3 at *qHT1.2* and *qHT6* may represent causal factors underlying the HT phenotype. Fine‐mapping, transgenic complementation, or CRISPR/Cas‐based knockouts could help determine whether OsSPL10, OsSPL11, and *pri‐miR156b/c* in *qHT1* and *qHT6* directly contribute to the HT phenotype.

Another distinctive feature of the HT phenotype is its temporal emergence after flowering. In contrast to classical tillering mutants, which typically affect early axillary meristem initiation during vegetative growth, HT plants exhibit repeated node formation and activation of new tillers during or after the reproductive transition. This delayed tiller emergence suggests that HT is not merely an enhancement of early tiller bud outgrowth but may involve altered regulation of meristem identity or vegetative‐to‐reproductive phase transition. Given that the *miR156–SPL* module plays a central role in phase transition and developmental timing, it is plausible that the natural allelic interactions identified in this study modulate this regulatory network in a stage‐specific manner. Therefore, the post‐flowering tillering pattern represents an important and biologically distinct aspect of the HT phenotype.

Although the *miR156–SPL* regulatory module has been extensively characterized in rice, our findings suggest that natural allelic variation can modulate this conserved pathway in a distinct developmental context. In previously reported cases, altered tillering was typically caused by transposon insertion or transgenic overexpression of *miR156* (Hayashi‐Tsugane et al., 2015). In contrast, the HT phenotype described here appears to result from the interaction of naturally occurring alleles at two independent loci. Thus, rather than identifying a new regulatory pathway, our study demonstrates how allelic interactions can rewire an existing developmental module to produce a novel plant architecture.

### 4.4 Transcriptomic profiling reveals extensive hormonal reprogramming

Transcriptomic profiling further elucidated the molecular landscape underlying the HT phenotype in HT35. DEGs revealed extensive alterations in photosynthesis, metabolic pathways, and hormone‐related processes (Figure 7). Notably, genes involved in the biosynthesis and signaling of GA, auxin, cytokinin, and other hormonal regulators displayed significant expression changes (Figure 8). Increased expression of GA biosynthetic genes such as *GA20ox* and *EUI1*, together with altered expression of GA signaling components including *SLR1* and *OsGAMYBL1*, is consistent with a potential involvement of GA‐related pathways in tiller development. Concurrently, differential expression of auxin‐ and cytokinin‐related genes, particularly members of the *OsIAA* and *OsCKX* families, may reflect multifaceted regulation of axillary meristem activity (Ashikari et al., 2005; Takeda et al., 2003).

Although extensive hormone‐related transcriptional reprogramming was observed, it remains unclear whether these changes represent primary drivers of the HT phenotype or secondary consequences of altered meristem development and tissue structure. Therefore, interpretation of hormone‐related DEGs should be considered provisional. Direct quantification of endogenous phytohormones during pre‐ and post‐flowering stages will be necessary to determine whether hormonal imbalance causally contributes to late‐stage tiller activation. In addition to enhanced tillering, certain secondary phenotypic features, including pseudo‐vivipary, were observed under winter greenhouse conditions but were not consistently detected in field environments. At present, the basis of this environmental sensitivity remains unclear. While the core post‐flowering tillering pattern was reproducibly observed across multiple years under field conditions, variation in phenotypic severity suggests that genotype × environment interactions may modulate specific aspects of HT expression. Further investigation will be required to clarify how environmental cues interact with the underlying genetic architecture to shape the full HT phenotype. The transcriptomic analysis presented here should be interpreted as complementary to genetic mapping results, providing insights into downstream transcriptional consequences of the HT architecture rather than definitive mechanistic proof.

Beyond transcriptomic observations, structural comparison with previously characterized mutants further highlights the distinct nature of HT. Previously reported higher‐order tillering mutants, such as *MOC1* overexpression lines and the grain number per panicle 6 (*gnp6*) mutant (Li et al., 2003; Zhang et al., 2021), exhibit altered tiller development but do not display repeated node formation as observed in HT. Moreover, *gnp6* mutants formed poorly developed panicles. In contrast, HT lines exhibit continuous tiller production from reiterated nodes and comparatively robust tiller development, supporting the notion that the underlying regulatory mechanism differs from previously characterized *MOC1*‐related mutants.

### 4.5 Implications and future perspectives for rice breeding

The combined QTL mapping and RNA‐seq analyses highlight extensive transcriptional reprogramming associated with HT and implicate candidate loci such as *qHT1* and *qHT6*, including *SPL*‐related genes, as key contributors to this phenotype. Notably, the HT trait emerged from an allelic interaction between cultivated rice and the weedy rice Hapcheonaengmi3, despite neither parental line exhibiting the phenotype. This finding illustrates that weedy rice harbors cryptic genetic variation that can give rise to novel plant architectures when introduced into a distinct genetic background.

Such emergent phenotypes, which are undetectable in parental lines but revealed through specific allelic combinations, underscore the importance of epistatic interactions in shaping complex agronomic traits. From a breeding perspective, this study highlights the potential value of underutilized germplasm as a reservoir of hidden alleles that may reconfigure conserved developmental pathways. Future studies integrating fine‐mapping, functional validation, and broader germplasm screening will be essential to determine the practical breeding utility of these HT loci and to explore whether similar cryptic interactions can be harnessed to optimize plant architecture and yield potential.

## AUTHOR CONTRIBUTIONS

**Kyu‐Chan Shim**: Conceptualization; data curation; visualization; writing—original draft; writing—review and editing. **DongHyun Jeon**: Data curation; visualization; writing—original draft. **Yun‐A Jeon**: Data curation; resources. **Cheryl Adeva**: Data curation; writing—review and editing. **Hyun‐Sook Lee**: Data curation; resources. **Ju‐Won Kang**: Resources. **Sa‐Eun Park**: Data curation. **Sang‐Nag Ahn**: Conceptualization; writing—review and editing. **Inkyu Park**: Conceptualization; writing—review and editing.

## CONFLICT OF INTEREST STATEMENT

The authors declare no conflicts of interest.

## Supporting information




**Figure S1**. Pedigree diagram of plant materials used in this study. * SSD: single seed descent, ** BILs: Backcross inbred lines, and *** ILs: introgression lines.
**Figure S2**. Comparison of rice plant morphology among two parental lines, Milyang23 and Hapcheonaengmi3, and their progeny HT37.
**Figure S3**. Plant morphology of HT35 which showed stronger high‐tillering phenotype and two parental lines.
**Figure S4**. Examination of high‐tillering stem and its continuous node development.
**Figure S5**. Examination of the pseudo‐vivipary phenotype in CR40 grown during the winter season.
**Figure S6**. Growth of high‐tillering stems and pseudo‐vivipary panicles in the soil, with seedlings emerging from the nodes and pseudo‐vivipary tissue.
**Figure S7**. Frequency distribution of high‐tillering phenotype in the 49 F_4:9_ population.
**Figure S8**. Sliding window analysis of delta SNP index with varying window sizes and increments. Delta SNP indices from QTL‐seq analysis were plotted along the *qHT1* region using different sliding window sizes and increments. As the window size and increment decreased (top left to bottom right).

**Table S1**. List of primer used in this study.
**Table S2**. High‐tillering phenotype data collected from F_4_ to F_9_ generation.
**Table S3**. Genome‐wide SNP dataset used for QTL‐seq analysis.
**Table S4**. Raw phenotypic scores and marker genotype data of the F_2_ population used for QTL validation on chromosomes 1 and 6.
**Table S5**. Candidate gene list of *qHT1.1* region.
**Table S6**. Candidate gene list of *qHT6* region.

**Supplementary Data S1**. Sliding Window Analysis Script (Python).

## Data Availability

The datasets supporting the conclusions of this article are included within the article and its additional files. Sequencing data used for QTL‐seq and RNA‐seq were deposited in the National Center for Biotechnology Information (NCBI) Sequence Read Archive (SRA) database under the BioProject accession number PRJNA1279397.
