# Supplementary material for: Genetic dissection and transcriptomic analysis of a novel high‐tillering phenotype in rice derived from weedy rice (Hapcheonaengmi3) and Tongil‐type Rice (Milyang23)
Source: Plant Genome. 2026 Apr 21;19(2):e70244. doi: 10.1002/tpg2.70244 (PMC13096764; doi:10.1002/tpg2.70244)
Supplement: Supplementary file 1 [file TPG2-19-e70244-s003.pdf]

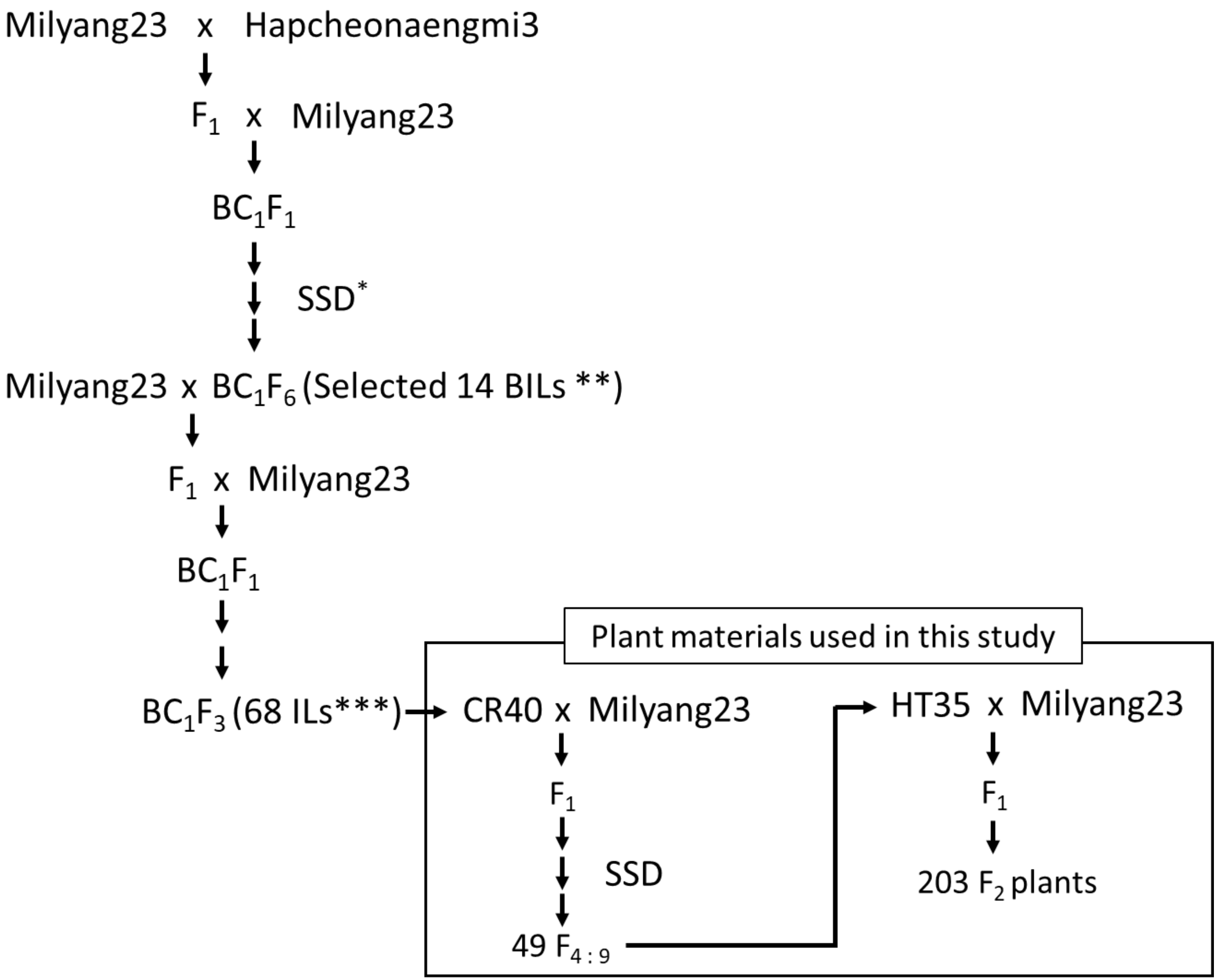

Fig. S1. Pedigree diagram of plant materials used in this study. * SSD: single seed descent, ** BILs: Backcross inbred lines, and *** ILs: introgression lines.

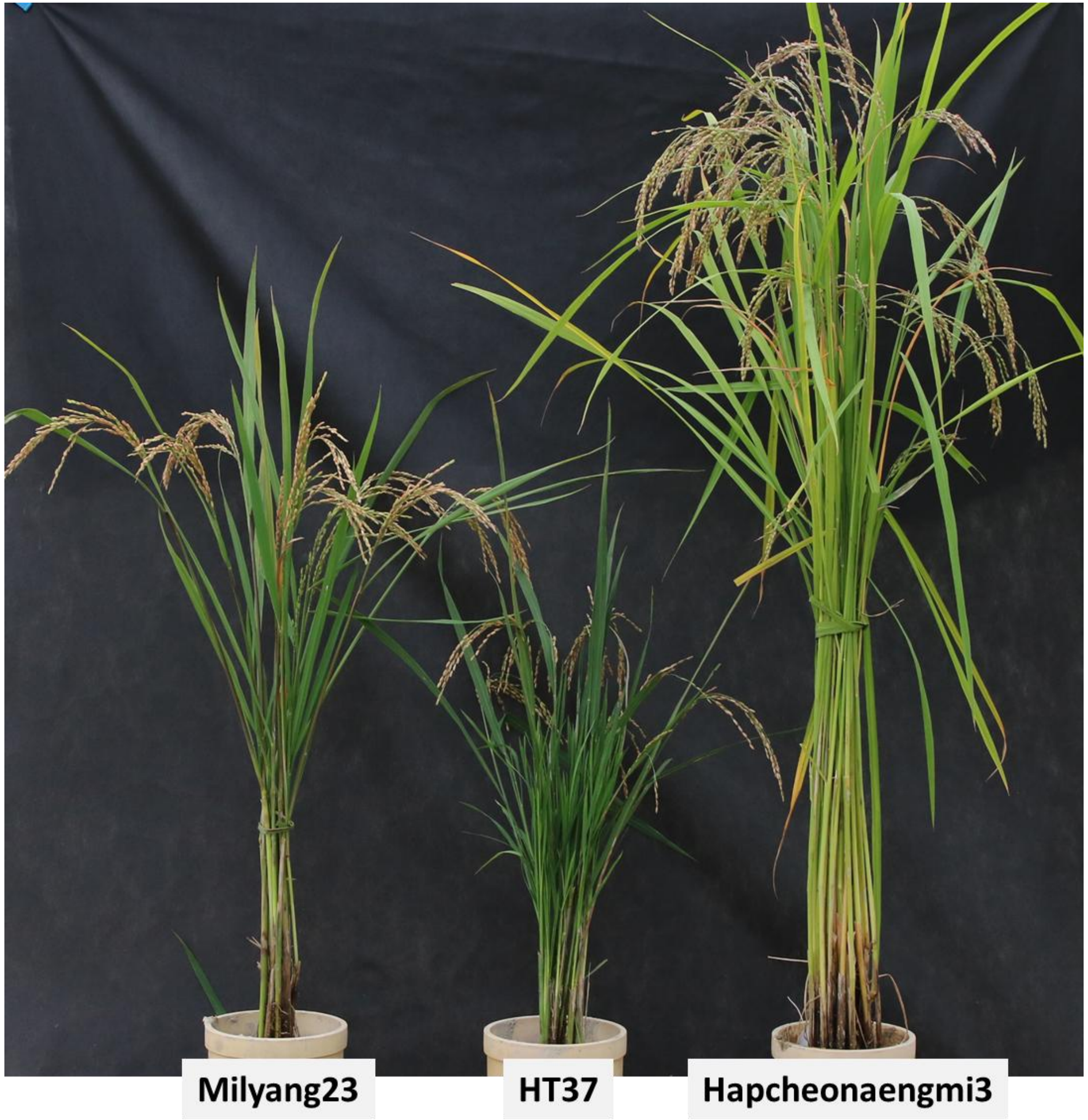

Fig. S2. Comparison of rice plant morphology among two parental lines, Milyang23 and Hapcheonaengmi3, and their progeny HT37.

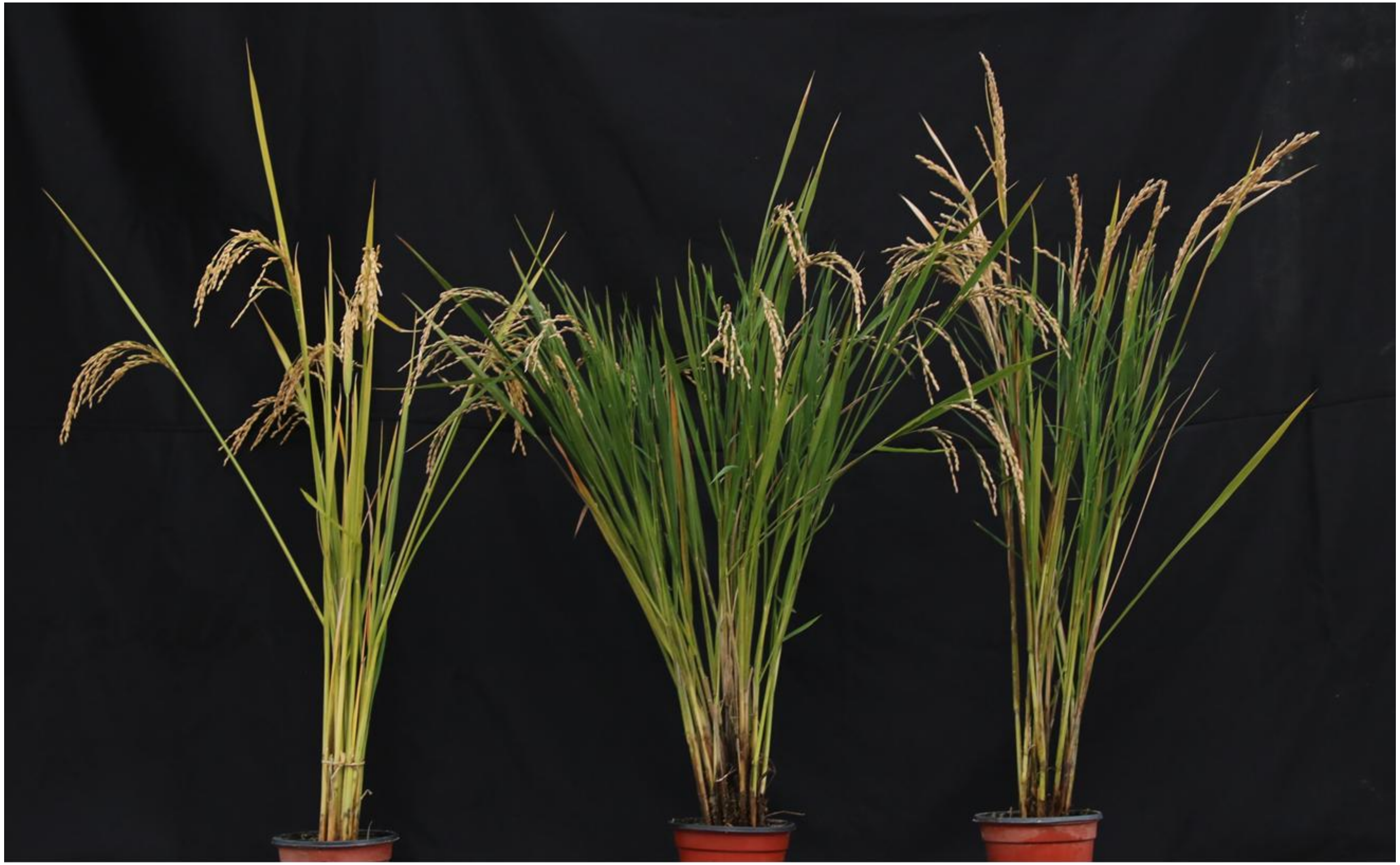

Milyang23 **HT35** **CR40**

Fig. S3. Plant morphology of HT35 which showed stronger high-tillering phenotype and two parental lines.

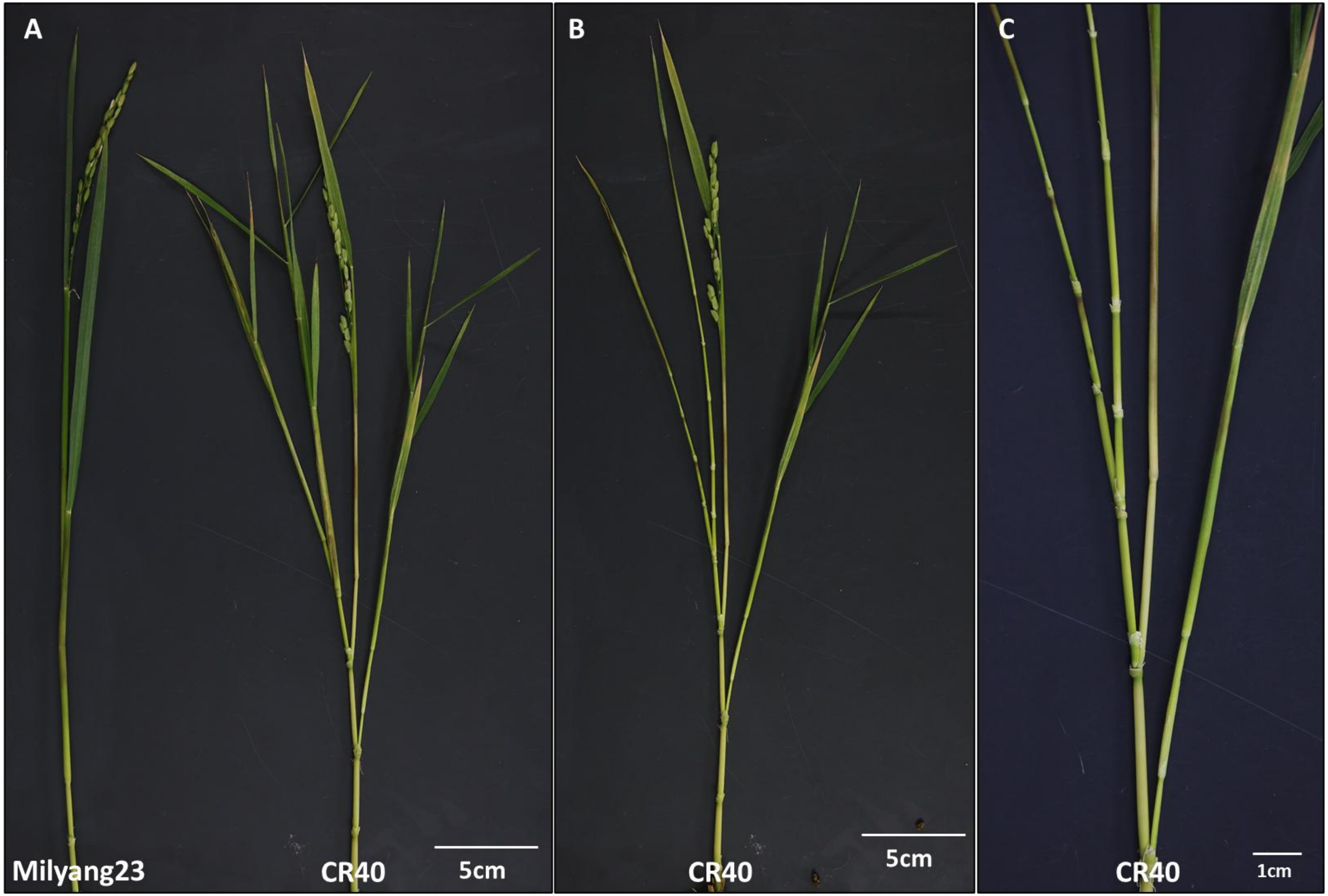

Fig. S4. Examination of high-tillering stem and its continuous node development.

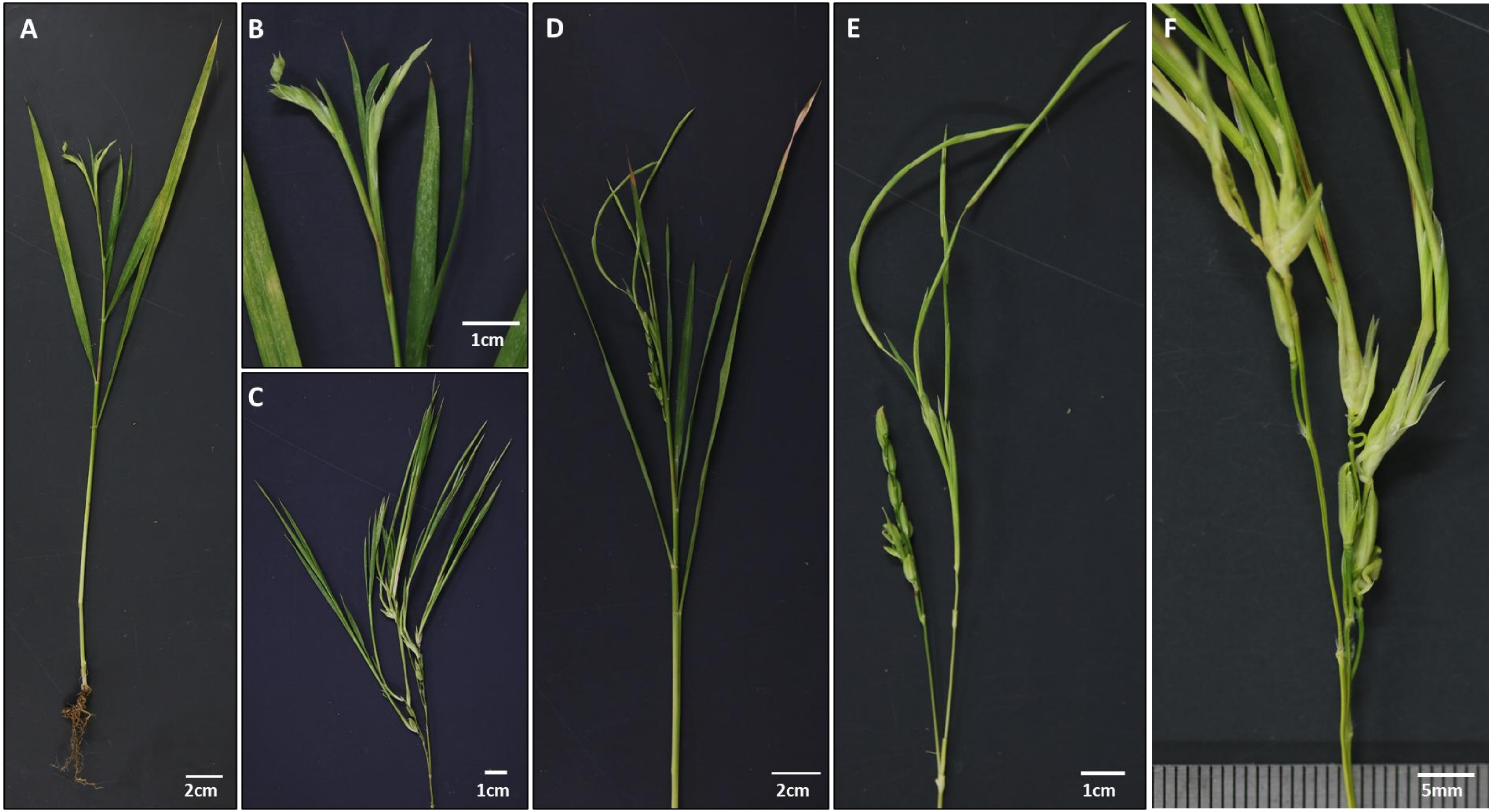

Fig. S5. Examination of the pseudo-vivipary phenotype in CR40 grown during the winter season.

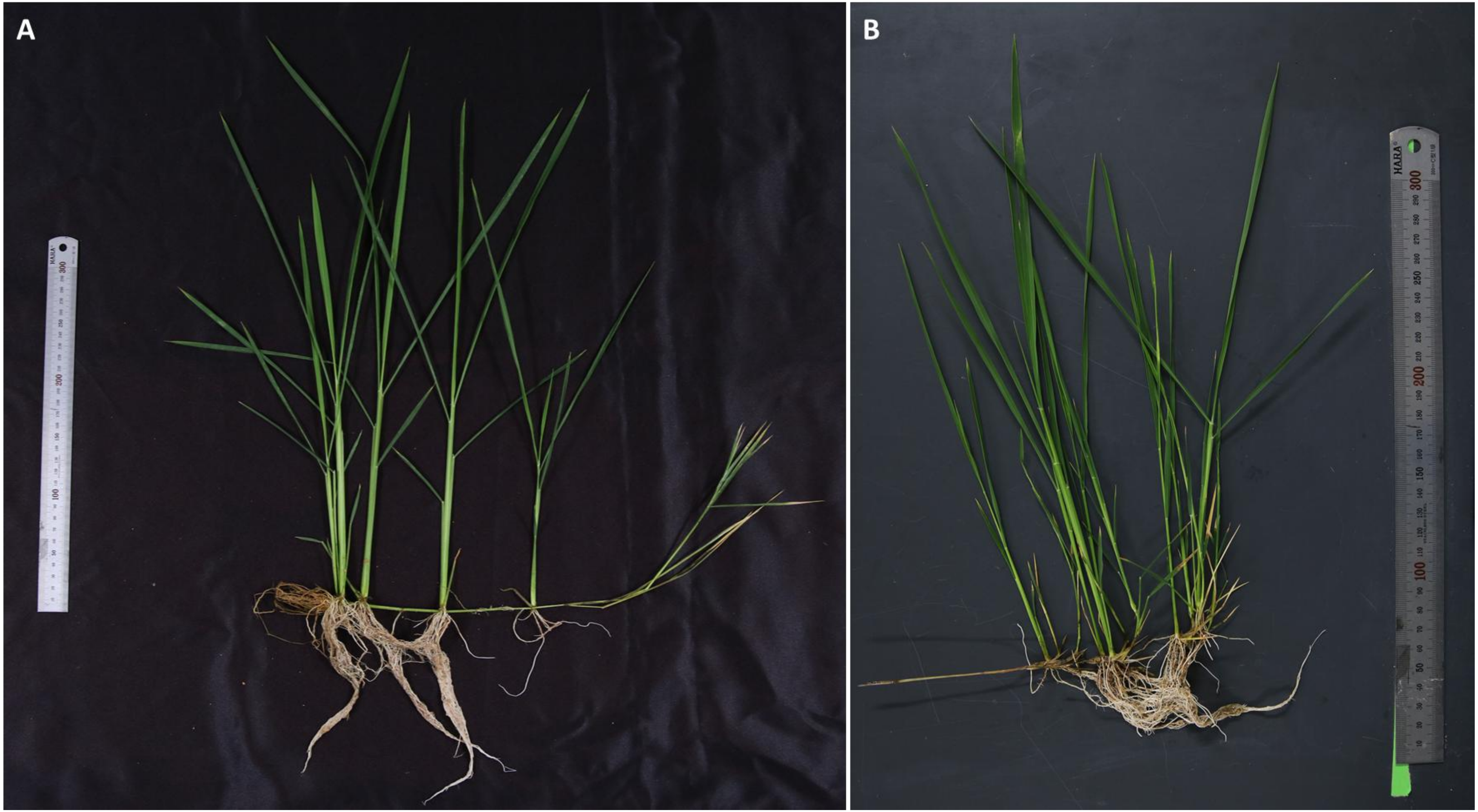

Fig. S6. Growth of high-tillering stems and pseudo-vivipary panicles in the soil, with seedlings emerging from the nodes and pseudo-vivipary tissue.

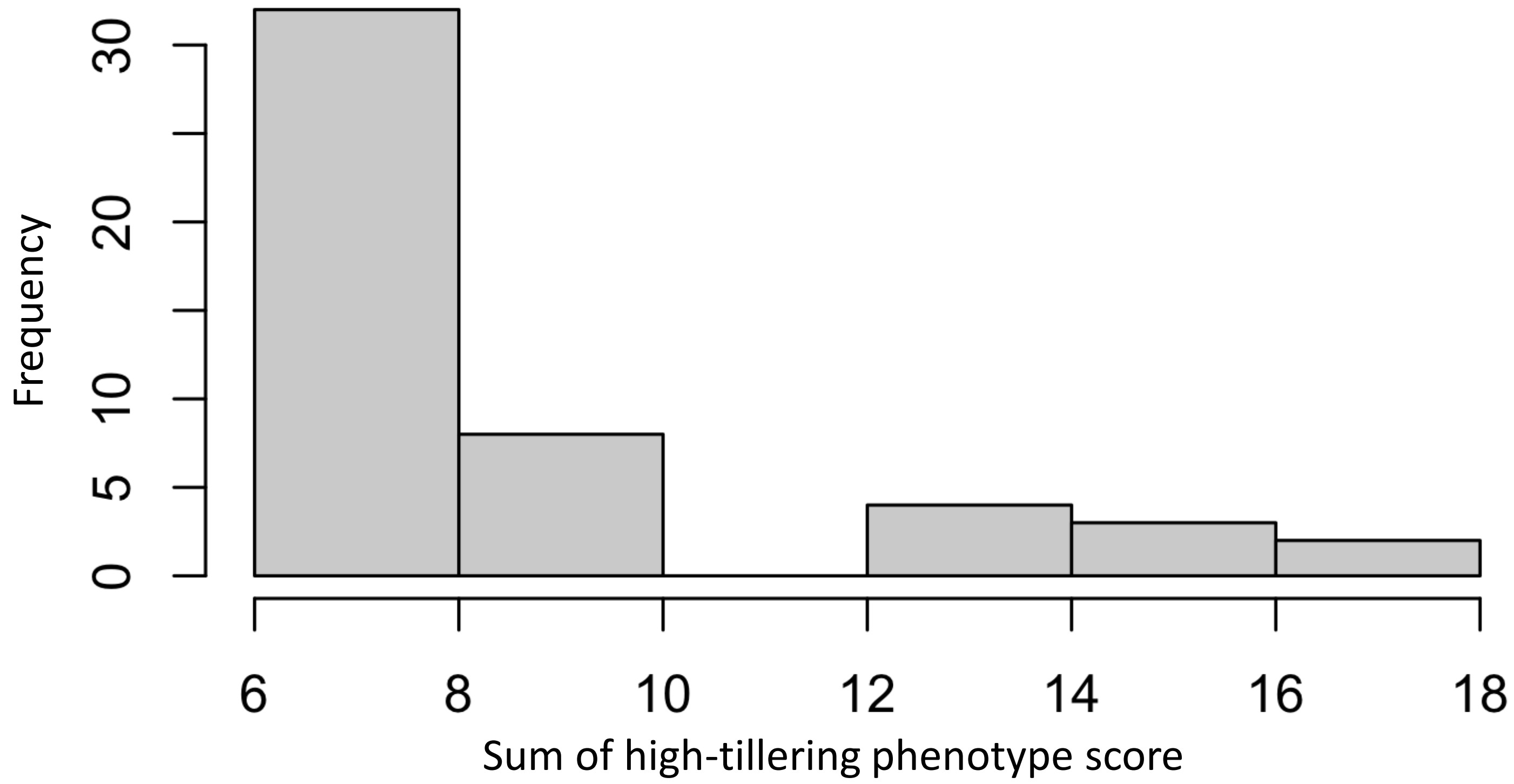

Fig. S7. Frequency distribution of the high-tillering phenotype in the 49 F4:7 population. The X-axis represents the sum of high-tillering phenotype scores measured over four years.

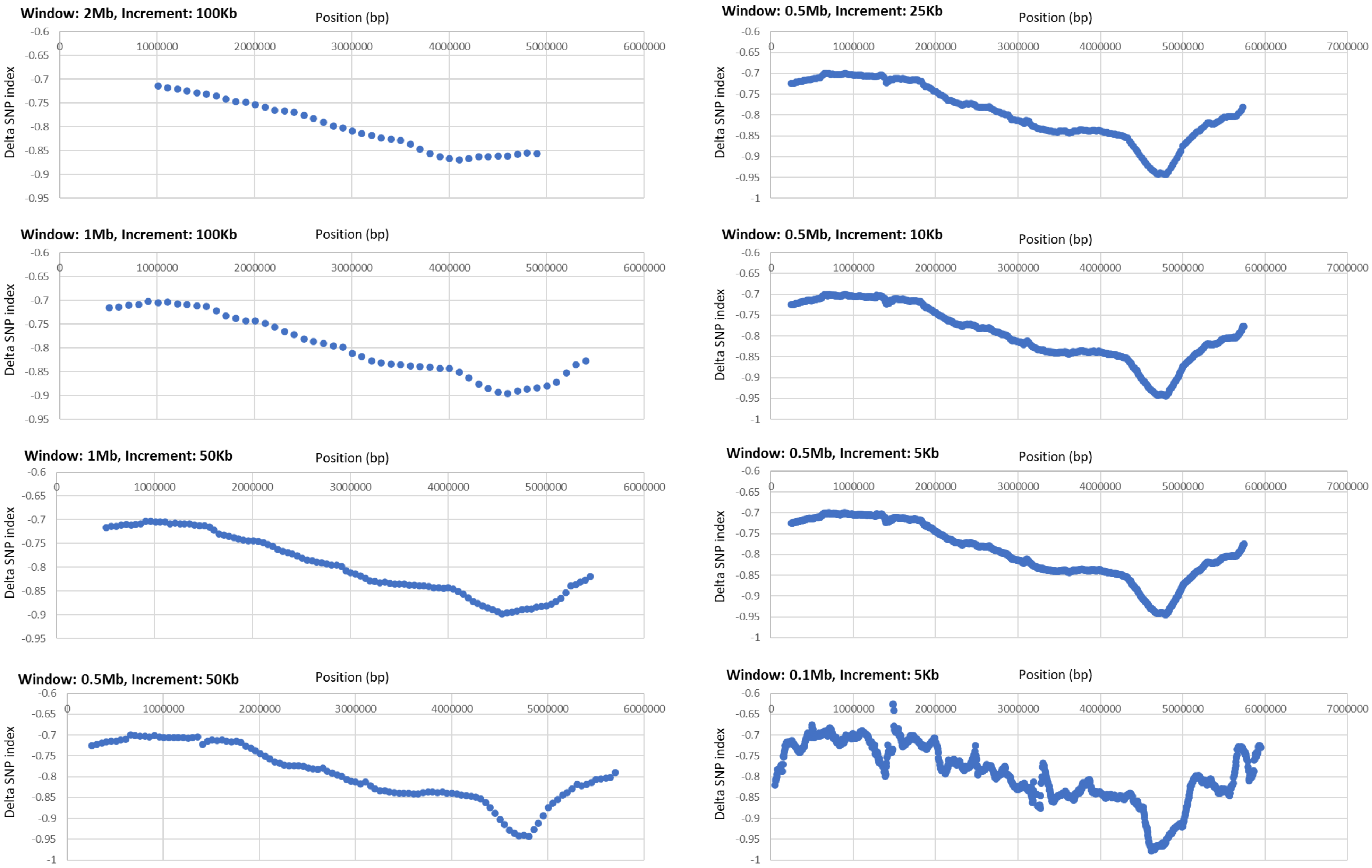

Fig. S8. Sliding window analysis of delta SNP index with varying window sizes and increments.
Delta SNP indices from QTL-seq analysis were plotted along the *qHT1* region using different sliding window sizes and increments. As the window size and increment decreased (top left to bottom right), the resolution increased with greater variability.
