# Supplementary material for: Genetic dissection and transcriptomic analysis of a novel high‐tillering phenotype in rice derived from weedy rice (Hapcheonaengmi3) and Tongil‐type Rice (Milyang23)
Source: Plant Genome. 2026 Apr 21;19(2):e70244. doi: 10.1002/tpg2.70244 (PMC13096764; doi:10.1002/tpg2.70244)
Supplement: Supplementary file 2 [file TPG2-19-e70244-s002.docx]

"""

Sliding Window Analysis Script

Supplementary Data S1

Description:

This script performs sliding window analysis for genomic data. It calculates the mean value

of a specified statistic (e.g., SNP index difference, FST) and the number of variants within each window.

Purpose:

This analysis was conducted to fine-map candidate regions identified by QTL-seq

for the high-tillering phenotype in rice.

Usage Notes:

- Input data should be a tab-delimited file (e.g., .tsv or .csv) containing at least

chromosome, position, and a statistical value (e.g., SNP index difference or FST).

- Only SNPs with statistically significant SNP index (p < 0.01) were included in this analysis

to reduce background noise and enhance resolution.

- Window sizes were tested from 2 Mb with 100 kb steps down to 10 kb with 5 kb steps.

The final analysis used a window size of 10 kb (0.01 Mb) and step size of 5 kb.

Author: Kyu-Chan Shim

Date: 2025.12.15

"""

import pandas as pd

def sliding_window(df, chrom_col, pos_col, value_col, window_size=10000, step_size=5000):

"""

Performs a sliding window analysis.

Parameters:

df (pd.DataFrame): Input dataframe with chromosome, position, and value columns.

chrom_col (str): Column name for chromosome.

pos_col (str): Column name for position (integer).

value_col (str): Column name for the value to average/count.

window_size (int): Size of the window in base pairs (default 10,000 = 10 kb).

step_size (int): Step size in base pairs (default 5,000 = 5 kb).

Returns:

pd.DataFrame: Dataframe containing sliding window results with columns:

['chrom', 'start', 'end', 'value_mean', 'count']

"""

results = []

df = df.sort_values(by=[chrom_col, pos_col])

for chrom in df[chrom_col].unique():

chrom_df = df[df[chrom_col] == chrom]

max_pos = chrom_df[pos_col].max()

start = 0

while start <= max_pos:

end = start + window_size

window_df = chrom_df[(chrom_df[pos_col] >= start) & (chrom_df[pos_col] < end)]

count = window_df.shape[0]

value_mean = window_df[value_col].mean() if count > 0 else 0

results.append([chrom, start, end, value_mean, count])

start += step_size

return pd.DataFrame(results, columns=['chrom', 'start', 'end', 'value_mean', 'count'])

\# Example usage:

\# df = pd.read_csv('filtered_snp_index.tsv', sep='\t') # e.g., with CHR, POS, SNP_index_diff columns

\# result = sliding_window(df, chrom_col='CHR', pos_col='POS', value_col='SNP_index_diff')

\# result.to_csv('sliding_window_result.tsv', sep='\t', index=False)
