# Supplementary material for: Genetic dissection and transcriptomic analysis of a novel high‐tillering phenotype in rice derived from weedy rice (Hapcheonaengmi3) and Tongil‐type Rice (Milyang23)
Source: Plant Genome. 2026 Apr 21;19:e70244. doi: 10.1002/tpg2.70244 (PMC13096764; doi:10.1002/tpg2.70244)
Supplement: Supplementary file 1 — Figure S1. Pedigree diagram of plant materials used in this study. * SSD: single seed descent, ** BILs: Backcross inbred lines, and *** ILs: introgression lines. Figure S2. Comparison of rice plant morphology among two parental lines, Milyang23 and Hapcheonaengmi3, and their progeny HT37. Figure S3. Plant morphology of HT35 which showed stronger high‐tillering phenotype and two parental lines. Figure S4. Examination of high‐tillering stem and its continuous node development. Figure S5. Examination of the pseudo‐vivipary phenotype in CR40 grown during the winter season. Figure S6. Growth of high‐tillering stems and pseudo‐vivipary panicles in the soil, with seedlings emerging from the nodes and pseudo‐vivipary tissue. Figure S7. Frequency distribution of high‐tillering phenotype in the 49 F4:9 population. Figure S8. Sliding window analysis of delta SNP index with varying window sizes and increments. Delta SNP indices from QTL‐seq analysis were plotted along the qHT1 region using different sliding window sizes and increments. As the window size and increment decreased (top left to bottom right). [file TPG2-19-e70244-s003.pdf]

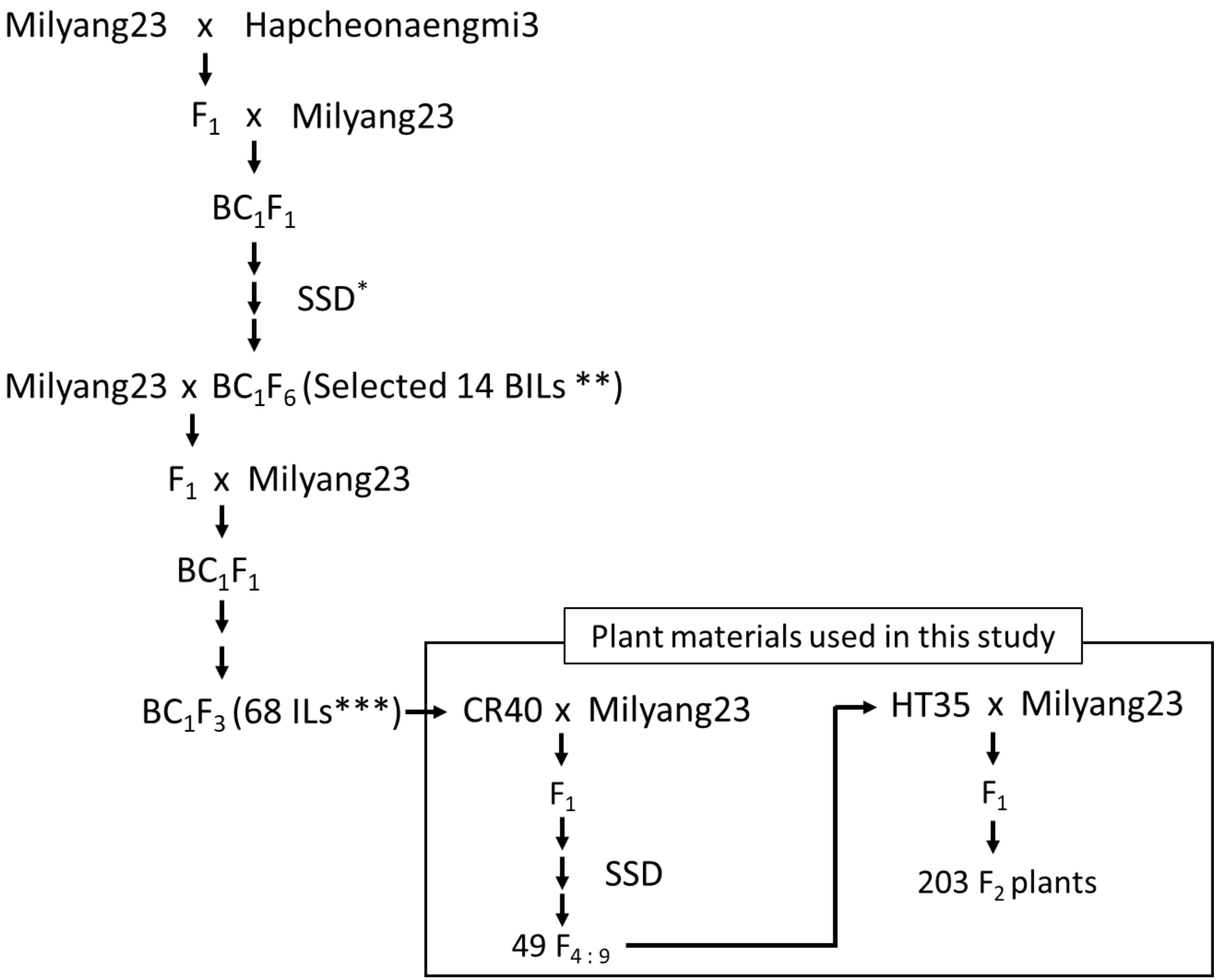

Fig. S1. Pedigree diagram of plant materials used in this study. * SSD: single seed descent, ** BILs: Backcross inbred lines, and *** ILs: introgression lines.

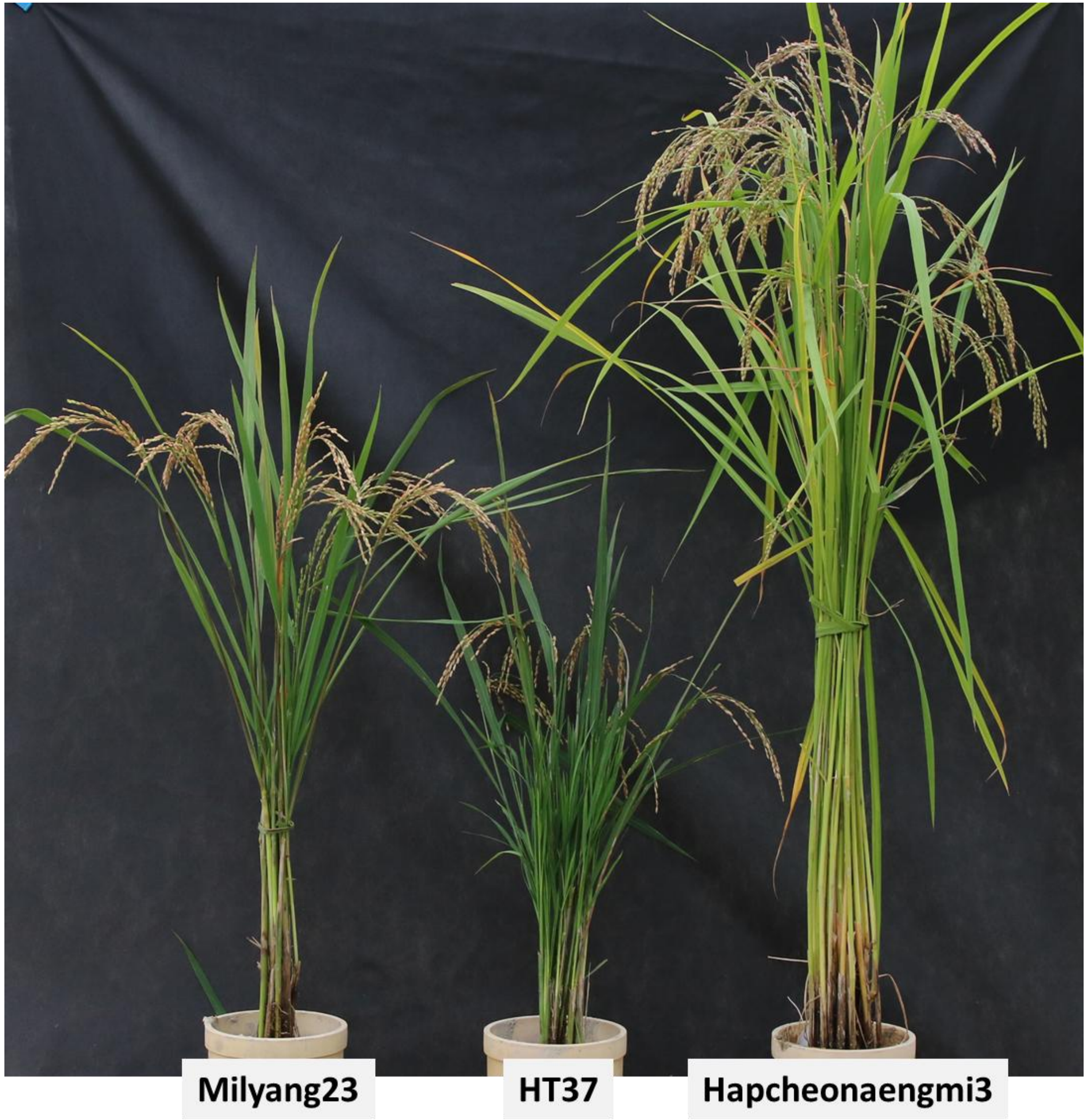

Fig. S2. Comparison of rice plant morphology among two parental lines, Milyang23 and Hapcheonaengmi3, and their progeny HT37.

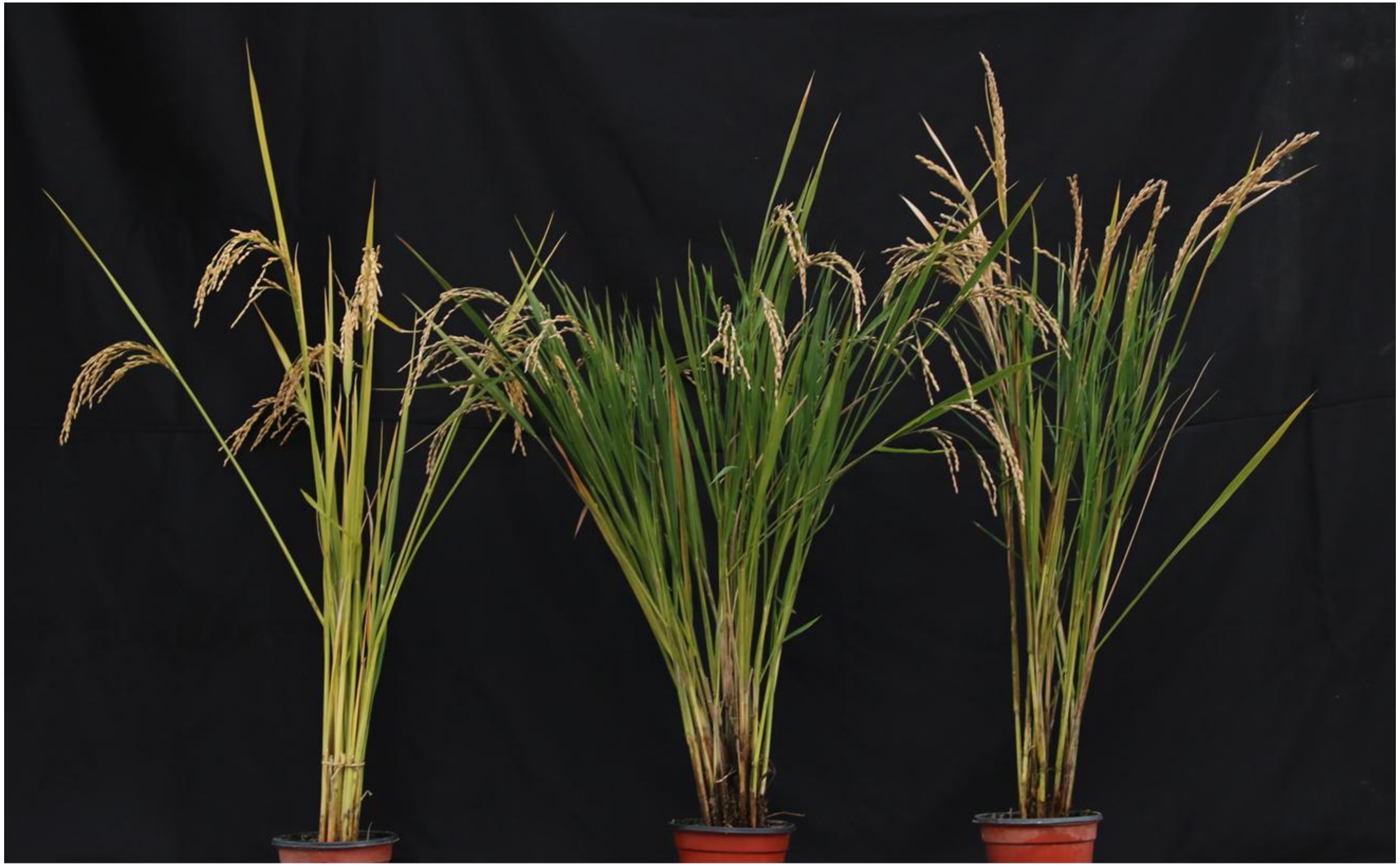

Milyang23 HT35 CR40

Fig. S3. Plant morphology of HT35 which showed stronger high-tillering phenotype and two parental lines.

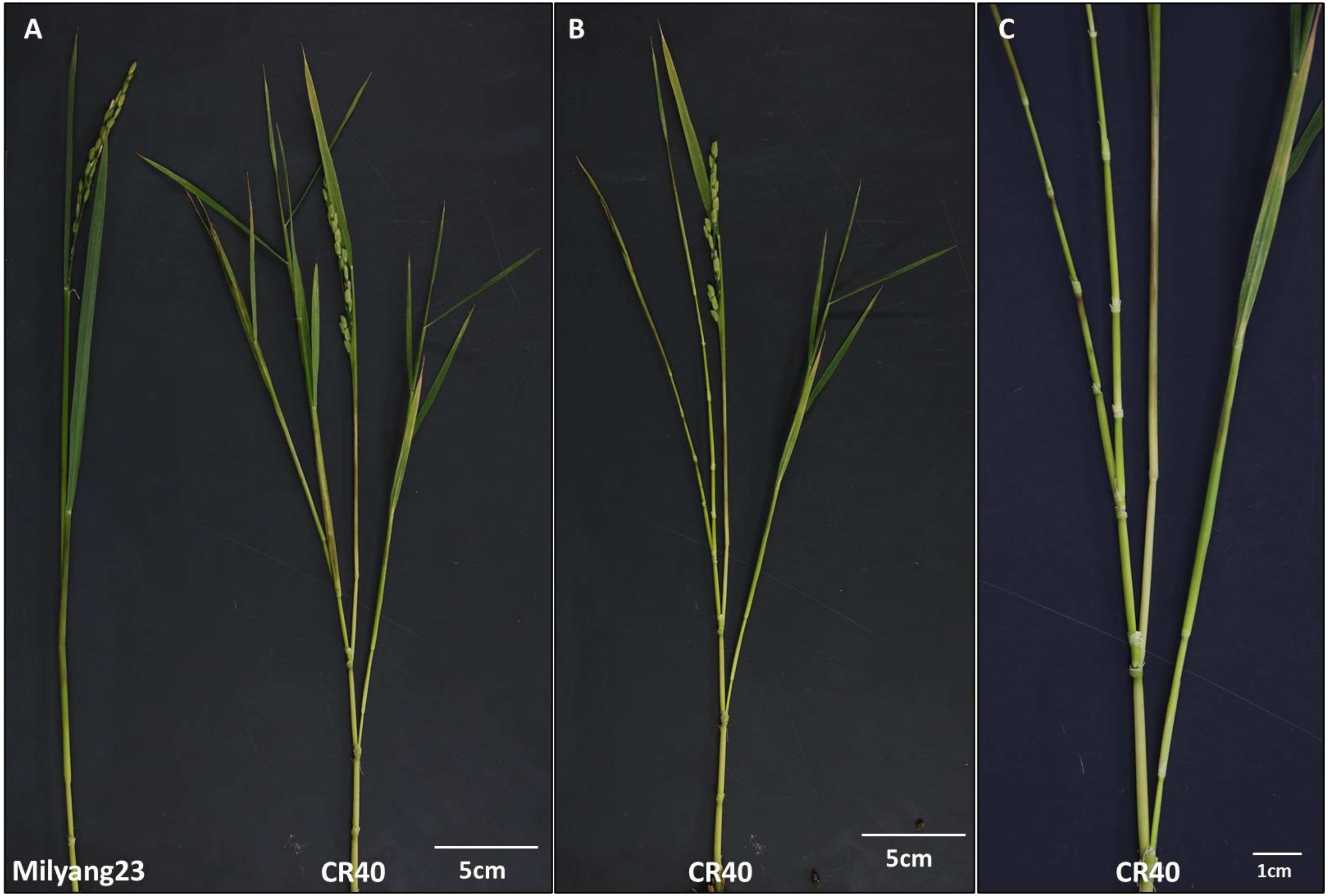

Fig. S4. Examination of high-tillering stem and its continuous node development.

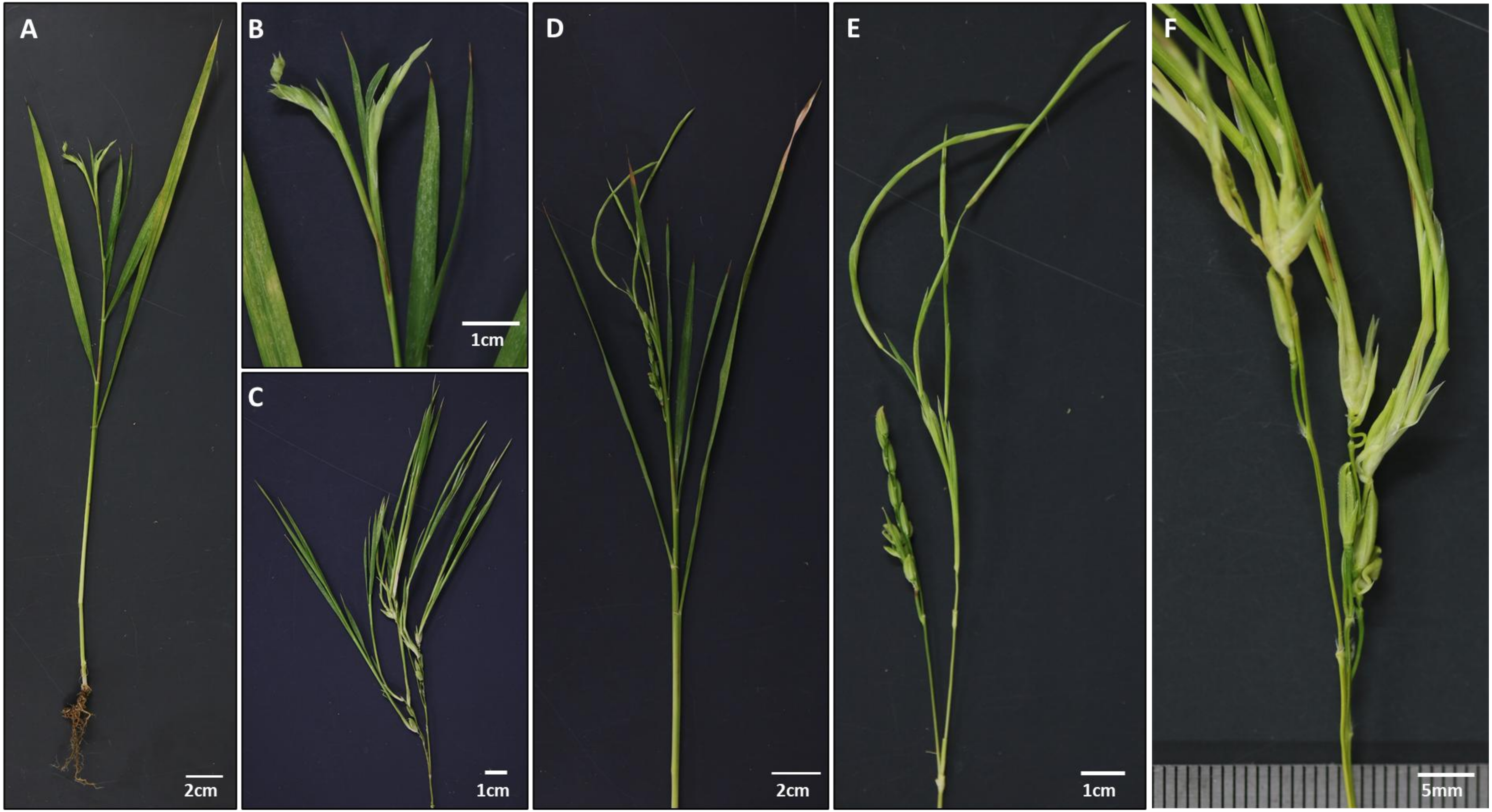

Fig. S5. Examination of the pseudo-vivipary phenotype in CR40 grown during the winter season.

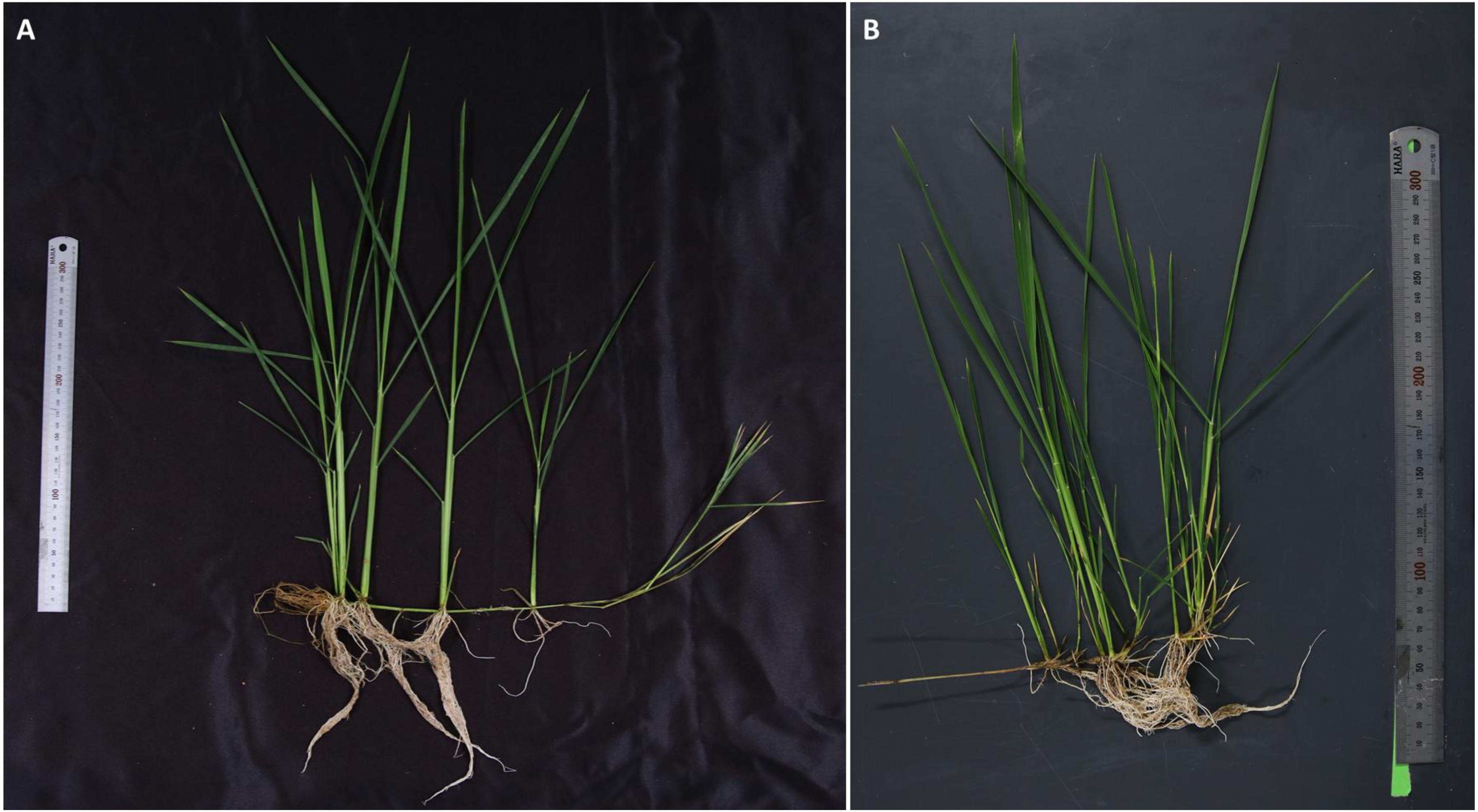

Fig. S6. Growth of high-tillering stems and pseudo-vivipary panicles in the soil, with seedlings emerging from the nodes and pseudo-vivipary tissue.

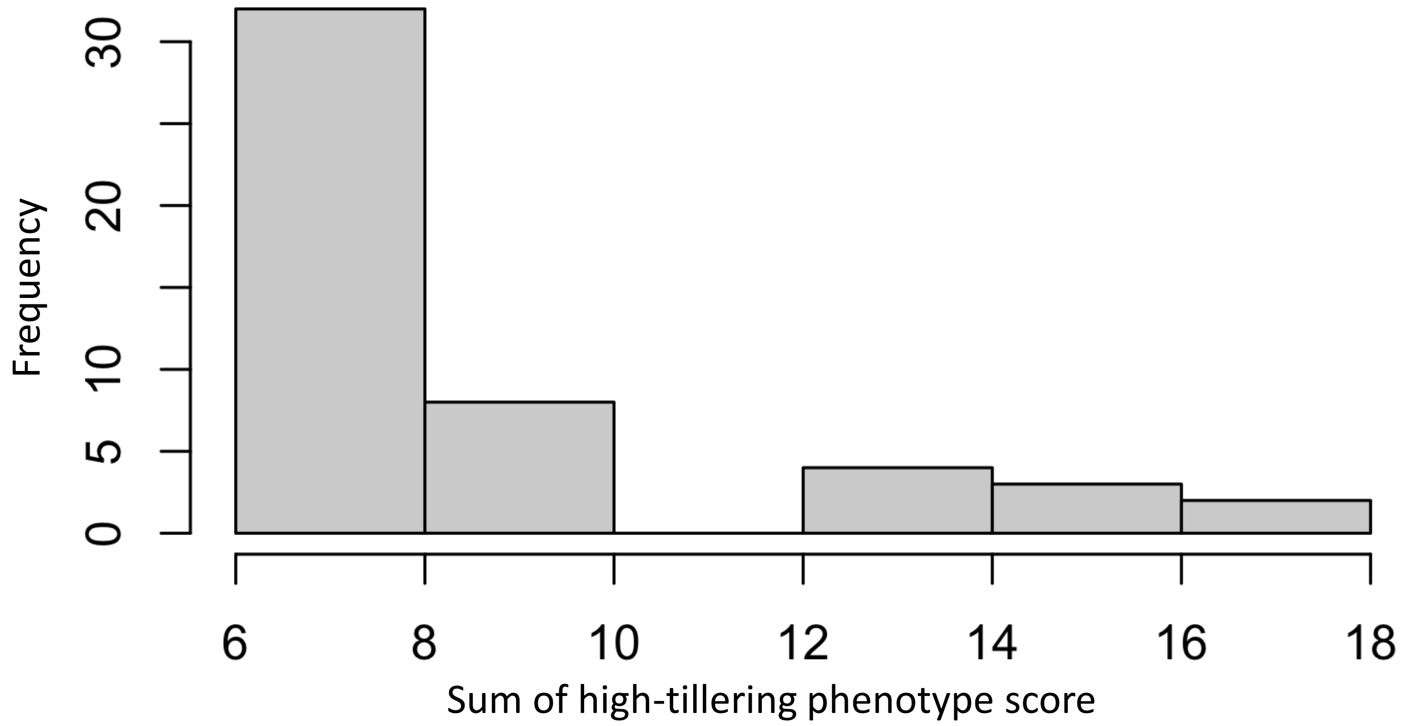

Fig. S7. Frequency distribution of the high-tillering phenotype in the 49 F4:7 population. The X-axis represents the sum of high-tillering phenotype scores measured over four years.

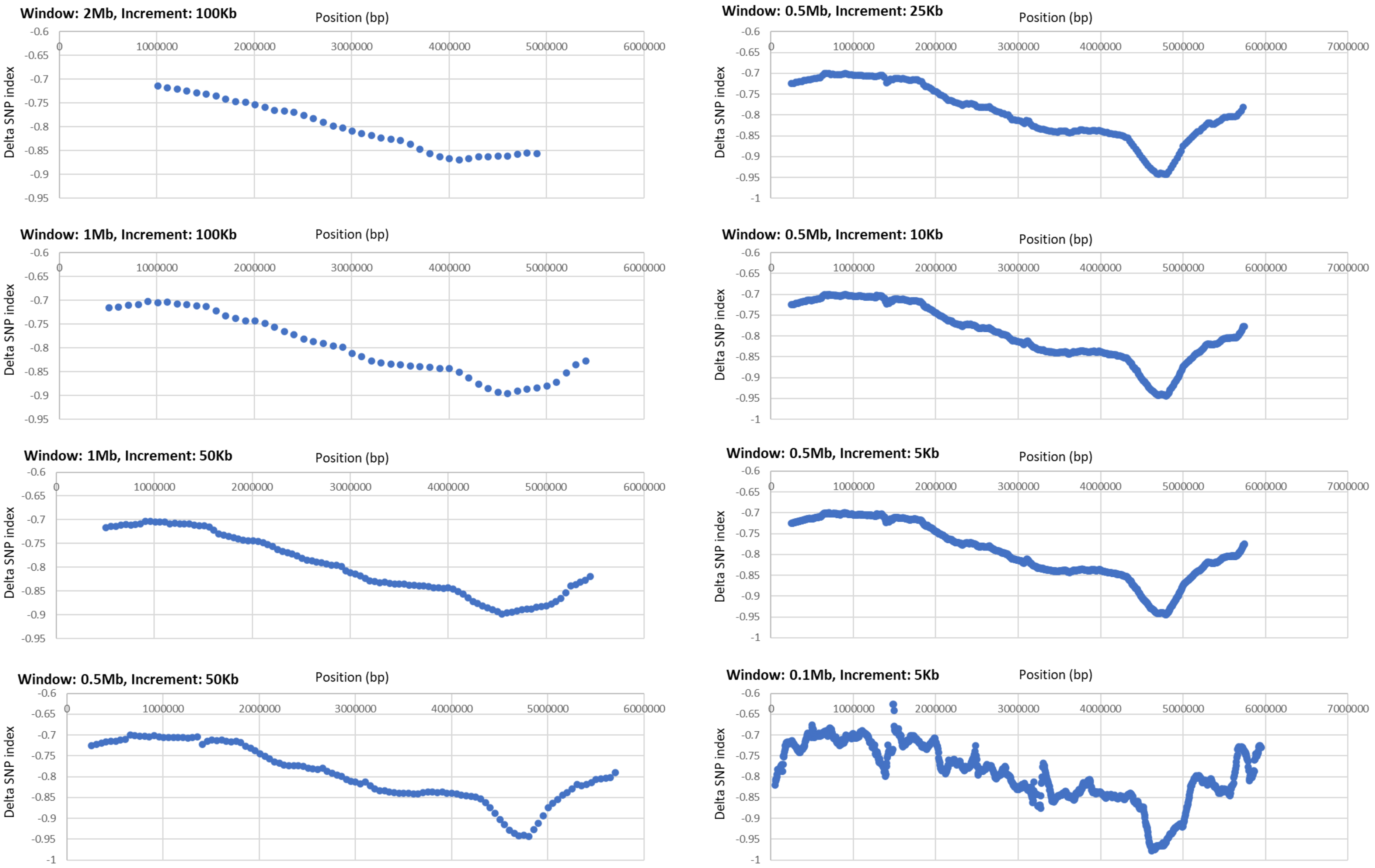

Fig. S8. Sliding window analysis of delta SNP index with varying window sizes and increments.
Delta SNP indices from QTL-seq analysis were plotted along the *qHT1* region using different sliding window sizes and increments. As the window size and increment decreased (top left to bottom right), the resolution increased with greater variability.
